# Naturally Derived Psilocybin for Therapeutic Use: A Six-Criterion Framework for Evidence, Safety, and Benefit–Risk Considerations in Policy and Clinical Development

**DOI:** 10.3390/biom16070983

**Published:** 2026-07-03

**Authors:** Stefanie Enriquez-Geppert, Lisa Bevers, Arvid Rosander, Peter Fodran, Vince Polito

**Affiliations:** 1 Department of Clinical and Developmental Neuropsychology, University of Groningen, 9712 CP Groningen, The Netherlands; 2 Department of Chemistry for Life Sciences, BMC, Uppsala University, 751 23 Uppsala, Sweden; 3 Department of Chemical and Pharmaceutical Biology, Groningen Research Institute of Pharmacy, University of Groningen, 9712 CP Groningen, The Netherlands; 4 School of Psychological Sciences, Macquarie University, Sydney, NSW 2109, Australia

**Keywords:** naturally derived psilocybin, therapeutic potential, safety and tolerability regulatory frameworks, pharmacological uniqueness

## Abstract

Naturally derived psilocybin is widely used, yet its therapeutic potential, pharmacological distinctiveness and regulatory feasibility remain understudied. This review evaluates the potential of naturally derived psilocybin using a six-criterion framework to evaluate: (1) therapeutic benefit, (2) safety and tolerability, (3) pharmacological uniqueness vs. synthetic psilocybin, (4) identity and composition control, (5) dose precision and stability, and (6) ecological sustainability. This paper answers three key questions about naturally derived psilocybin: Does it show therapeutic potential? Does it differ from synthetic psilocybin? Can it meet medicinal standards? Findings suggest perceived therapeutic benefits from naturally derived psilocybin across mental health domains, though evidence of causal efficacy is mixed. Safety profiles are favorable but context-dependent, with risks in vulnerable populations. Some preliminary preclinical evidence indicates possible entourage effects, but human validation is lacking. Dose precision varies, with purified psilocybin being most reliable, followed by standardized extracts, alcoholic, aqueous, and whole biomass preparations. Scalable cultivation is feasible but faces sustainability challenges. Key gaps include a lack of controlled trials, longitudinal safety evaluations, and standardization. We provide a phased research roadmap, which proposes short-term studies to establish safety, mid-term mechanistic and standardization efforts, and long-term integration into therapeutic, cultural, and ecological systems. This review highlights the promise of naturally derived psilocybin but underscores the need for rigorous evidence to support regulatory acceptance and clinical use.

## 1. Introduction

Psychedelic research has re-emerged as a clinically relevant field at the intersection of psychiatry, psychology, and neuroscience, showing promise as a treatment for depression, addiction and existential distress (e.g., [1,2,3,4]). Several countries have now authorized restricted therapeutic use of psychedelics through regulated medical access pathways [5,6], and low and middle-income countries have also demonstrated readiness to implement these therapies [7]. Among currently investigated psychedelic substances, psilocybin has a number of important features: it is relatively well studied with strong signs of clinical utility; it has a long history of use in various cultures around the world [8]; and high rates of naturalistic use [9].

Notably, naturally derived psilocybin has functioned for centuries as a medicine within Indigenous ceremonial healing systems, particularly in Mesoamerica. These systems embed psilocybin with a diagnostic, relational, spiritual, political, and cosmological framework. Historically, psilocybin mushrooms were used for analgesic, anxiolytic, divinatory, and “soul-healing” purposes [10,11,12,13].

Psilocybin entered Western biomedical discourse following R. Gordon Wasson’s account of his participation in a Mazatec traditional healing ceremony in Mexico [14]. This exposure led Albert Hofmann to isolate and synthesize psilocybin, which was subsequently distributed to clinicians for experimental use. This pathway from ethnobotanical use to biomedical research has been identified as one of the most productive strategies for drug discovery. Fabricant and Farnsworth [15] found that 80% of clinically used plant-derived compounds had ethnomedical uses that aligned with their modern indications, underscoring the value of traditional knowledge not only for identifying active compounds, but also for informing their therapeutic framing.

Despite the dual trajectory of increased clinical research into psychedelics and longstanding cultural use, the therapeutic potential of naturally derived psilocybin remains largely unexamined in contemporary research. While synthetic psilocybin dominates clinical trials, there is little formal evidence regarding the therapeutic potential, contextual relevance, or unique characteristics of natural preparations. This review addresses this gap by asking three guiding questions:

(T) Do naturally derived psilocybin preparations demonstrate therapeutic potential in human populations (in real-world use)? (T: therapeutic potential). This is a foundational question; without therapeutic benefit, other considerations become secondary.

(S) Do naturally derived psilocybin preparations produce pharmacological or experiential effects that are meaningfully distinct from synthetic psilocybin? (S: specificity compared to synthetic psilocybin). This question evaluates whether the form of psilocybin meaningfully impacts outcome, experience, or safety.

(MP) Do naturally derived psilocybin preparations meet criteria for consideration as therapeutic agents or regulated medicinal products? (MP: medical product viability)

These questions structure the review’s analytic framework and are reflected across six evaluation criteria, enabling an integrated assessment of naturally derived psilocybin’s current and potential value in mental health and well-being.

## 2. Methods

### 2.1. Conceptual Foundations

Contemporary clinical psychology and psychiatry adopt a biopsychosocial framework, defining mental health as more than the absence of illness, instead emphasizing a dynamic balance across biological, psychological, and social domains [16,17,18]. The World Health Organization [19] describes mental health as “a state of well-being in which an individual realizes their own abilities, can cope with the normal stresses of life, can work productively and fruitfully, and is able to contribute to their community”. Within this spectrum, mental health and mental illness exist on a spectrum, with mental well-being representing the positive end.

In this biopsychosocial framework, treatment refers to evidence-based interventions targeting mental illness or dysfunction, whereas therapy encompasses structured, often relational processes that support recovery, self-understanding, and personal growth beyond symptom alleviation. Therapeutic potential describes an intervention’s ability to improve mental health, whether by reducing symptoms, alleviating suffering, or enhancing well-being, resilience and existential flourishing [20,21,22,23,24].

In this review, the term “naturally derived psilocybin” is used collectively to refer to psilocybin obtained from *Psilocybe* mushrooms, truffles, and mycelium (or unpurified whole-mushroom extracts). Importantly, “naturally derived psilocybin” contains additional bioactive compounds as described in Section 3.4. Conversely, “synthetic psilocybin” refers to the same molecular entity as that found in nature, but produced through chemical synthesis or extracted in a pure form from mushrooms without any additional bioactive compounds.

### 2.2. The Six-Criterion Framework to Evaluate Naturally Derived Psilocybin

To rigorously assess our three guiding questions, we developed a six-criterion framework to evaluate the merits of naturally derived psilocybin. This framework is grounded in regulatory and consensus evaluation guidelines, including CONSORT-Herbal (reporting guidelines to improve transparency and quality of clinical trials involving plant-based or natural products), World Health Organization guidelines on Good Agricultural and Collection Practices for medical plants (WHO GACP), European Medicines Agency (EMA) Committee on Herbal Medicinal Products guidelines for herbal medicinal products (EMA HMPC), and US Food and Drug Administration guidelines for industry regarding botanical drug development and biological products [25,26].

The evaluation criteria are:

(1) Therapeutic benefit: Is there evidence that naturally derived psilocybin produces measurable benefits in humans?
(2) Safety and tolerability: Can naturally derived psilocybin be used without undue risk, and under what conditions?
(3) Psychopharmacological uniqueness: Does natural psilocybin offer pharmacodynamic or experiential effects distinct from synthetic forms?
(4) Identity and composition control: Can naturally derived psilocybin be authenticated, standardized, and verified as contaminant-free?
(5) Dose precision and stability: Are potency and effects consistent across preparations and time?
(6) Ecological sustainability: Can natural sourcing or cultivation meet demand without causing environmental or cultural harm?

This six-criterion framework serves as the basis for our evaluation.

### 2.3. Search Strategy

This study employed a narrative non-systematic review methodology. Relevant literature was identified through searches of electronic databases and integrates human studies, animal models and pharmacological data alongside ethnographic accounts. Both formal evidence (e.g., laboratory studies, controlled cohort studies) and real-world evidence (RWE; e.g., population surveys, ethnographic work, qualitative analyses) are included. RWE may be particularly relevant for evaluating naturally derived psilocybin as regulatory agencies are increasingly incorporating RWE alongside clinical trials (e.g., [25,26,27,28,29]; see Table 1). No formal systematic search protocol was followed. The following sections apply this six-criterion framework to the available evidence, with emphasis on the three guiding questions.

## 3. Results

### 3.1. Background Context for the Evaluation

#### 3.1.1. Global Prevalence: Distribution and Cultural Prevalence of Naturally Derived Psilocybin Use Worldwide

Analyses of US National Survey on Drug Use and Health (NSDUH) data indicate that psilocybin use is widespread. Using 2005–2014 data (*n* = 381,682), Jones et al. [9] reported lifetime psilocybin use of 11.4% among white Americans, 4.9% among Hispanics, and 3.1% among other participants of color. This is lower than lysergic acid diethylamide LSD (32.1%) but higher than most other illicit substances. Later NSDUH waves (2015–2018; n = 168,650) analyzed by Yockey and King [33] showed increasing prevalence and a strong education gradient, with higher use among college-educated individuals. These data demonstrate substantial naturalistic exposure, providing a real-world foundation for assessing human therapeutic outcomes in Criterion 1. However, these estimates derive exclusively from US samples and may not generalize to other cultural contexts.

Winstock et al. [34] analyzed large-scale international data of the Global Drug Survey (GDS), which provides valuable ecological insight into real-world data from GDS 2021 with data of 32,022 respondents from 22 countries to assess how COVID-19 changed drug use behaviors, including also alcohol, cannabis, and cocaine. Of the total sample, 33.4% used psilocybin mushrooms ever, and 15.7% in the last year. Among those who use psychedelics, microdosing in particular seems to be on the increase, with one in four psychedelic users reporting microdosing with LSD or psilocybin in the last few months.

#### 3.1.2. Use Patterns and Motivations: Common Reasons for Use and How These Influence Consumption Practices

Lake and Lucas [35] analyzed responses from GDS data with 6379 self-selected psychedelic users across 85 countries, predominantly from Anglophone regions. Psilocybin emerged as the most commonly used psychedelic (reported by 90.8% of participants) across all regions. Most participants reported infrequent psychedelic use, typically once every one to five months, and around 80% reported past-year use. Motivations were diverse: personal growth (~85%), enhancement of general well-being (~69%), and recreation (~60%) were cited most often. Medical or self-treatment motives were more common in North America and Australia/New Zealand (~43%) than in Europe/UK (35%) or other regions (~30%). About 16% of users indicated using psychedelics to reduce other substance use, suggesting potential harm-reduction applications. Although most users currently obtain psychedelics from friends (~56%) or dealers (~32%), there was a strong preference for regulated and autonomous forms of access, such as self-production or harvesting, retail dispensaries, and pharmacies or clinics, particularly in Europe/UK and Australia/New Zealand. Regional variations were evident: users in Canada and the U.S. reported more frequent microdosing, therapeutic intentions, and ibogaine use, whereas European users expressed stronger preferences for self-cultivation and pharmacy-based access.

In a prospective naturalistic study by Nayak et al. [36] 2833 participants reported self-exploration (~81%), mental health (~71%), therapy (~50%), creativity (~44%), recreation (~38%), productivity (~22%), and physical health (~14%) as their motivation for their psilocybin experience.

Across these studies, the prominence of well-being as a primary motive is particularly relevant to treatment readiness. As conceptualized by Ruggeri et al. [37], well-being encompasses physical, psychological, and social functioning, positive emotions, and realization of potential, extending beyond the absence of illness and linked to productivity, learning, creativity, and prosocial behavior. Frequent self-reported psilocybin use for well-being, therefore, suggests a growing demand for interventions that aim not only at symptom reduction but also at fostering positive mental health and psychological flourishing.

Overall, these global findings highlight key aspects of naturally derived psilocybin use: the salience of well-being as a legitimate health goal, the persistence of self-treatment motives, and consumer preferences pointing toward decentralized, accessible, and potentially self-directed therapeutic models. These reported motives underscore the importance of examining whether naturally derived psilocybin use is, in fact, associated with measurable therapeutic benefits in humans, as evaluated in Criterion 1.

#### 3.1.3. Consumption Settings: Variability in Use, Solo vs. Group vs. Organized Retreats, and How These Contexts Impact Dosing, Preparation, and User Experience

The way naturally derived psilocybin is used varies across cultures and user populations. In general, there are no population studies or large-scale studies providing prevalence data on solo vs. non-solo usage of psychedelics. However, the naturalistic prospective study by Nayak [36] revealed that most participants (who were white and male from the US) used psilocybin alone (43.0%) or with friends who were also using it (25.7%), while 16.4% had a sober friend as a sitter. A small percentage used it with a shaman, guide, or therapist (each under 2%). The majority consumed psilocybin at home (69.7%) or in nature (15.8%), with fewer using it in spiritual, social, or public settings (each under 3%).

Retreats, though less prevalent than solo use, are notable for their structured, intentional frameworks, and research also reveals growing numbers. For instance, Neitzke-Spruill et al. [38] mapped 298 psychedelic retreat organizations active in 2023, demonstrating the rapid global expansion of retreat-based use of natural psychedelics. Most retreats offered ayahuasca (73.8%), followed by psilocybin (25.5%) and San Pedro (20.1%), and the majority positioned themselves as wellness-oriented (90.5%) rather than clinical. U.S. retreats were more likely to adopt an explicitly religious framing (55% vs. ~1% elsewhere), whereas medically framed models were rare. Many organizations invoked Indigenous lineages or “ancestral wisdom” as part of their branding, particularly when offering psilocybin. Retreats were located across 440 sites globally and spanned a wide price range.

The demand for guided psilocybin experiences extends beyond retreats, giving rise to another phenomenon: the emergence of underground facilitators who guide psychedelic sessions, often self-taught, and operate outside regulated frameworks. While there is no exact prevalence data, qualitative studies reveal that this community is diverse, but seems to share a commitment to harm reduction, personal growth, and the therapeutic potential of psilocybin [39,40].

### 3.2. Criterion 1: Human Evidence of Therapeutic Benefit

This criterion assesses documented evidence of therapeutic evidence from naturally derived psilocybin, defined by the biopsychosocial model: improvements in biological functioning (e.g., physiological stress or substance use reduction), psychological outcomes (e.g., mood, anxiety, coping, insight), and/or social/functional outcomes (e.g., relationships, role functioning, participation, community integration).

Evidence is drawn from observational and naturalistic studies (surveys, longitudinal cohorts, retreat-based studies), early phase experimental/feasibility studies, and ethnographic case material from Indigenous or ceremonial contexts.

This section is organized by study design, clarifying how each methodology contributes to understanding therapeutic benefits. Given the predominance of observational/qualitative studies, findings are interpreted within the Evidence-Based Medicine (EBM) hierarchy, balancing methodological rigor and strength of causal inference.

#### 3.2.1. Observational Evidence

##### Population-Based Surveys

Large epidemiological datasets provide direct real-world evidence of health-related associations with psilocybin use. Hendricks et al. [41], analyzing data from nearly 200,000 adults in the National Survey on Drug Use and Health (NSDUH), found that lifetime psilocybin use, unlike other classic psychedelics, was particularly associated with lower levels of past-month psychological distress. Psychological distress is a broad symptom cluster reflecting difficulties coping with stressors, linked to increased risk of chronic disease, work disability, and sickness absence [42].

In a separate NSDUH analysis, Jones et al. [43] found reduced odds of past-year hypertension among non-Hispanic white psychedelic users, though this association was not observed in other racial/ethnic groups. Pooled NSDUH studies of classic psychedelics show that lifetime psychedelic use is associated with reduced likelihood of psychological distress (−19%), suicidal thinking (−14%), suicidal planning (−29%), and suicide attempts (−36%) compared to individuals with no reported lifetime use of psychedelics [44]. By contrast, lifetime use of other illicit substances (e.g., cocaine, stimulants, opioids) was typically associated with elevated risks of psychological distress and suicidality [43,45,46,47].

In contrast to lifetime-use analyses, investigations of past-year psilocybin use have reported somewhat different health-related associations. Matzopoulos et al. [48] conducted a survey of 7139 US adults, weighted to represent the national adult population for past-year psilocybin mushroom use. Within this sample, 1.7% reported consuming only psilocybin in the past year (i.e., no other drugs). If this rate generalized to the entire US population, this would equate to 4.1 million adults (out of a total population of 233 million). Results indicated that individuals who had consumed psilocybin in the past year reported significantly higher rates of depression, anxiety, migraines, and insomnia, as well as lower mental health scores and health-related quality of life compared with non-users. Despite these burdens, psilocybin mushroom users expressed more favorable views of their potential for mental health benefits, including for general well-being, personal development, and psychiatric conditions. Motives for use were most frequently general mental health and well-being (~64%), followed by treatment of diagnosed psychiatric conditions (~32%) and self-diagnosed concerns (~19%). Psilocybin mushroom users were also more likely than non-users to report noticing increased positive media coverage of psychedelics for mental health in the previous six months. In terms of healthcare utilization, psilocybin mushroom users showed higher rates of urgent care visits (21% vs. 10%) and hospitalizations (9% vs. 4%) than non-users, with ~18% reporting that they had sought medical treatment following use. Together, these findings illustrate the dual profile of US psilocybin mushroom users: a group with elevated mental health burden and healthcare use, yet simultaneously holding optimistic views and motives for psilocybin mushrooms as tools for well-being and mental health support.

Overall, population-level studies in the US suggest that psilocybin and other classical psychedelics are often associated with positive effects on mental health, such as reduced psychological distress and suicidality. However, these associations are not consistent across all outcomes or demographic groups, and the cross-sectional nature of the data precludes causal conclusions. Notably, positive effects (e.g., lower risk of high blood pressure) were observed primarily among non-Hispanic white participants. One possible explanation for this is that structural inequalities, higher exposure to chronic stress, and cultural context may obscure or attenuate potential benefits in minority populations. This underscores the need for further research that incorporates social determinants of health to ensure culturally informed and equitable psychedelic research. This discrepancy between lifetime use and past-year use may reflect differences in populations captured: lifetime-use analyses may include many individuals who experimented occasionally and experienced long-term resilience, whereas past-year use may over-represent those with current mental health needs who turn to psilocybin as self-treatment. Taken together, these findings highlight that population-level associations depend on the timeframe and groups sampled, and reinforce the importance of considering both social determinants of health and patterns of use when interpreting outcomes.

##### Large-Scale International Online Surveys

Glynos et al. [49] investigated naturalistic psychedelic use for chronic pain in a sample of 466 individuals from the GDS 2023 who reported attempting to self-treat a chronic pain condition. Many participants reported reduced substance use, improvements in both physical and mental health symptoms, and decreased reliance on conventional mental health treatments. Among those who reduced or stopped at least one non-psychedelic substance (n = 391), psilocybin was most frequently cited as the most effective catalyst (29.9%), followed by ketamine (12.5%) and ayahuasca (11.8%). Psilocybin also ranked highest among those who reduced or ceased prescription opioid use (33.3%, n = 33/100) and illicit opioid use (18.8%, n = 9/48) following psychedelic use. Moreover, psilocybin was the most commonly endorsed substance for improving both physical health symptoms (29.4%, n = 137/466) and mental health conditions (48.9%, n = 133/270). These self-reported outcomes point to the perceived therapeutic value of psilocybin in treating complex, comorbid conditions such as chronic pain, particularly its potential to reduce opioid use and conventional pharmacological burden. Although these results come from uncontrolled, self-selected samples, they provide ecologically valid data that warrant further investigation in controlled clinical settings.

Kopra et al. [50] analyzed data from the GDS 2020 to investigate self-treatment of psychedelic use. Of 113,234 respondents, 10,268 (14%) reported using psychedelics in the past year to address psychiatric conditions or psychological concerns, most commonly depression (~40%), anxiety (~20%), and relationship problems (~9%). Among these, 3328 used LSD and 2394 psilocybin mushrooms for this purpose, and 1996 and 1368 indicated LSD and psilocybin-producing mushrooms as the most useful substances. Self-treatment outcomes were assessed using a 17-item self-treatment outcome scale measuring changes in well-being, symptoms, social–emotional functioning, and health behavior. On average, respondents reported mild to moderate improvements across all domains, with roughly half experiencing above-average benefits on at least half the items. Effects typically lasted beyond three weeks. Psilocybin use, treatment of post-traumatic stress disorder (PTSD), seeking advice beforehand, and high-intensity experiences were associated with more positive outcomes. In contrast, negative outcomes were more likely among younger users, those self-treating with LSD, and those reporting high-intensity experiences. These findings suggest that naturally derived psilocybin may be perceived by users as a relatively effective and manageable self-treatment option, especially for mood and trauma-related symptoms, while also highlighting key risk factors that could guide harm-reduction and inform future clinical frameworks.

Morton et al. [51] conducted an international web-based survey of 542 individuals with self-reported bipolar disorder who had used psilocybin. About one-third reported adverse effects, most often manic symptoms, sleep disturbances, and anxiety. Most respondents reported achieving their intended goals for psilocybin use, including curiosity, mental health treatment, personal development, spiritual growth, interpersonal bonding, and cognitive enhancement. Of the 314 participants (58% of the sample) who responded to the open-ended question about positive experiences with psilocybin, 214 described positive effects, including improved mood, well-being, and personal insight. These findings highlight both the risks and potential benefits of psilocybin in bipolar populations and underscore the need for controlled studies in this vulnerable group.

##### Prospective Naturalistic Studies

Jones et al. [52] conducted a longitudinal online survey (n = 2833) with assessments before psilocybin use, 2–4 weeks afterward, and again at 2–3 months, comparing outcomes between white participants and participants of color. Psilocybin use was associated with reductions in depression and anxiety and improvements in spiritual well-being and cognitive flexibility across groups. However, effects diverged over time: spiritual well-being and cognitive flexibility gains persisted at follow-up for white participants but attenuated among participants of color. These findings suggest that psilocybin can support positive mental health outcomes, yet the durability and magnitude of benefits are moderated by ethnic background, underscoring the influence of contextual and structural factors such as cultural meaning, access to supportive settings, and chronic stress.

In a large naturalistic longitudinal study, Nayak et al. [36] tracked the experiences of 2833 individuals before and after using psilocybin. Participants were primarily highly educated white men from the US, with a mean age of 40 years. Results showed a persistent reduction in anxiety, depression and alcohol misuse and neuroticism, as well as increased cognitive flexibility, emotion regulation, spiritual well-being and extraversion.

In order to improve prediction of acute and longer-term responses to psychedelics, Haijen et al. [53] tracked 212 psychedelic users across five timepoints (48% reporting psilocybin use). They found that well-being increased up to four weeks post-use, especially when experiences involved mystical-type features, clear intentions, and a positive mindset. By contrast, recreational intentions were linked to more challenging effects. Baseline traits (e.g., absorption) and dose level further shaped acute experiences and subsequent well-being.

##### Observational Studies of Psilocybin Retreats

There is an emerging research literature investigating the effects of psilocybin retreats. In a prospective observational study, Kettner et al. [54] assessed acute relational experiences of perceived togetherness and shared humanity in 886 participants, of which 80% used naturally derived psilocybin. Results demonstrated that acute experiences of “communitas” (i.e., a profound sense of collective unity) during a psychedelic ceremony are primarily driven by perceived emotional support and pre-session rapport with both group members and facilitators. Although communitas during the ceremony correlated with positive outcomes, enduring psychological benefits were fully mediated by the extension of communitas beyond the acute experience, particularly through self-disclosure and group sharing practices (e.g., sharing rounds). These findings underscore the critical role of social–environmental factors, including trust, emotional openness, and structured communal interactions, in amplifying and sustaining the therapeutic potential of collective psychedelic use.

Qualitative and pilot studies give further insight into participant experiences during retreat settings: Lutkajtis [55] qualitatively examined four individuals’ experiences during and after a psilocybin truffle retreat in the Netherlands. Participants reported variable but often mystical-type experiences, with shifts in self-perception and a strong sense of connectedness to self, others, and the wider world. Narratives emphasized moments of key insight and embodiment as a central theme. Pilecki et al. [56] conducted a pilot study at a Jamaican psilocybin retreat where nine participants consumed between 2 and 5 g dried psilocybin mushrooms. Large and durable improvements were observed in psychological flexibility, with reductions in cognitive fusion and an increase in self-compassion, maintained up to six months. Participants showed rapid decreases in barriers to valued living (e.g., rumination or disengagement) and increases in alignment with personal values over time.

These findings point to potential mechanisms through which naturally derived psilocybin retreats foster lasting improvements in mental health, aligning with treatment models that emphasize psychological flexibility and self-compassion as transdiagnostic therapeutic targets.

Microdosing with naturally derived Psilocybin: motivations, practices and outcomes

Psilocybin microdosing refers to the repeated use of sub-perceptual doses over time, a practice reported to have distinct effects and risks compared to high-dose use (see review [57]).

Petranker et al. [58] analyzed GDS 2019 data, which included more than 123,000 respondents and 2832 psilocybin microdosers. While the majority reported microdosing with LSD, psilocybin was the second most common substance. Reported benefits from microdosing included improved mood, focus, and creativity. The most frequently reported challenge was “none at all”, with more than half experiencing no adverse effects. When difficulties were noted, they most often involved physiological discomfort, impaired energy (restlessness/fatigue), or cognitive interference (e.g., confusion, memory issues, racing thoughts).

Rootman et al. [59] surveyed 8703 respondents from 84 countries, including 3486 psilocybin microdosers and 447 LSD microdosers, and compared them with non-microdosers. Psilocybin was the most common substance (>85%), typically taken 1–4 times per week. Users often combine (“stack”) psilocybin with lion’s mane (39%), niacin (18%), or chocolate (5%). Microdosers reported lower anxiety and depression than non-microdosers and endorsed broad health-related motivations, especially mindfulness, mood, creativity, and learning. Nearly one-third reported mental health or substance-use concerns (mainly anxiety, depression, PTSD/trauma, tobacco dependence). Microdosers were more likely than non-microdosers to endorse depression, PTSD/trauma, and tobacco dependence, but not anxiety or alcohol/cannabis problems. Among those with mental health concerns, motives centered on reducing anxiety, decreasing substance use, and improving mood.

Rootman et al. [60] extended this work with a prospective online survey of 953 psilocybin microdosers and 180 controls, with follow-up at 25–30 days. Microdosers showed small- to medium-sized improvements in mood and mental health, consistent across age, gender, and baseline concerns, as well as enhanced psychomotor performance, particularly in older adults. Stacking psilocybin with lion’s mane and niacin did not affect mood or mental health, but in older adults, this combination was linked to greater psychomotor improvements.

Together, these studies suggest that microdosing with naturally derived psilocybin may confer modest benefits for mood, well-being, and cognitive performance, though findings are limited by observational designs, self-selection, expectancy, and other nonspecific influences.

Perceived effectiveness of psilocybin microdosing compared to regular doses and conventional treatments

Hutten et al. [61] surveyed 410 microdosers with prior full-dose experience to compare the perceived effectiveness of microdosing, conventional treatments, and regular psychedelic doses for mental and physiological conditions. Respondents reported 901 mental and 161 physiological diagnoses, most commonly depression (73%), anxiety (56%), attention deficit hyperactivity disorder (ADHD; 37%), and, on the physiological side, migraines (15%) and chronic pain (14%). Psilocybin was the predominant substance in both microdosing (57%) and regular dosing (65%) contexts. Effectiveness ratings indicated that microdosing was perceived as more effective than conventional treatments, particularly for ADHD and anxiety. However, regular psychedelic doses were rated more effective than microdosing for depression and anxiety, while no difference emerged for physiological disorders. Similarly, Lea et al. [62] reported that amongst individuals prescribed psychiatric medications, 50.6% ceased antidepressants and 39.7% ceased other medications after commencing microdosing.

These findings position psilocybin as a common microdosing substance for self-treatment, with perceived advantages over conventional therapies, though its effectiveness has not been well tested in rigorous controlled trials.

Naturally derived psilocybin microdosing and ADHD

Haijen et al. [63] reported on two prospective naturalistic studies with adults with severe ADHD symptoms, most of whom microdosed psilocybin-containing mushrooms or truffles (with a minority using novel lysergamides, LSD, or ayahuasca). In their first study (*n* = 233), they found improvements in emotion regulation and aspects of empathy in this patient sample. In their second study (*n* = 180), they compared microdosers with adults receiving conventional ADHD medication. After 4 weeks, microdosers reported lower ADHD symptom severity across all subscales, falling below the clinical threshold, whereas the medication group improved only on inattention. No significant differences emerged for empathy. Together, these findings suggest that psilocybin microdosing may improve ADHD symptoms and emotion regulation compared with conventional medication. However, despite this promising signal, a recent RCT from the same group failed to find evidence that LSD microdosing improved ADHD symptoms [64].

#### 3.2.2. Ethnographic Documentation: Contemporary Indigenous Use

The terms healing, cure, and medicine are used in the following to reflect Indigenous and holistic frameworks, where healing encompasses restoration of coherence and harmony across mind, body, spirit, and community, and cure is the complete resolution of illness. Contemporary ethnography confirms continuous cultural use of psilocybin among Mazatec, Chatino, Chinantec, Mixe, Zapotec, Nãjã, and Matlatzinca groups, with the Mazatec most extensively documented [8,65,66,67,68]. Outside Mesoamerica, culturally anchored psilocybin healing is also documented among Basotho healers in Lesotho [69] and in Igala communities in Nigeria [70], indicating that community-embedded psilocybin medicine is geographically diverse. Transdisciplinary initiatives such as the Dialog of Knowledge project examine naturally derived psilocybin within its original use of context, which includes the spiritual and cultural practices [71].

Fagetti and Mercadillo [72] documented Mazatec veladas (tsakjena kón ka’oña, “staying awake together”): nocturnal psilocybin ceremonies embedded in preparatory and post-ingestion rituals (abstinence, silence, and ritual purity). Testimonies describe spiritual diagnosis, symbolic extractions (e.g., vomiting), reconciliation of social tensions, and healing rooted in Catholic-Indigenous cosmologies, such as a participant’s eye affliction, unresolved biomedically, interpreted as sorcery and healed through visions of Catholic Virgins extracting a “thorn”, restoring sight and instructing prayer. Illness here reflects socially embedded spiritual imbalance: the soul-essence (*ase’a*) or dreaming spirit (*shimajo*) may be displaced or attacked, requiring ritual visionary journeys to restore vitality. Psilocybin acts not as an isolated drug, but as a divine agent enabling diagnosis and healing within a ritual–cosmological system [73].

These examples underscore that psilocybin’s therapeutic role extends beyond pharmacology, embedding healing in social, spiritual, and communal dimensions. Recognizing these broader contexts and meanings can contribute to culturally sensitive approaches to explore the therapeutic potential of naturally derived psilocybin, always adapted to the belief systems, values, and needs of participants, and always in harmony with respect for their origins.

##### Proposed and Early Phase Indigenous-Anchored Research

While most contemporary therapeutic applications of psilocybin are developed within Western biomedical paradigms, a small number of initiatives are beginning to explore Indigenous-led or Indigenous-informed models within regulated or clinical contexts.

For instance, Hodge et al. [74] present the Tū Wairua Project, a Māori-led health initiative in Aotearoa (New Zealand) that integrates rongoā Māori (traditional healing) with psilocybin-assisted therapy to address methamphetamine misuse. This open-label mixed-method study is being conducted on a “marae”, a sacred communal space, to preserve cultural integrity and ancestral connection. Quantitative measures include validated anxiety scales, Māori-specific wellness assessments (Te Whare Tapa Whā), and physiological safety indices; qualitative data are collected via semi-structured interviews. Naturally derived psilocybin will be administered as 25 mg GMP-grade whole-mushroom preparations, chosen over synthetic compounds to retain *mauri* (vital essence) and connection to *Papatūānuku* (Earth Mother). Grounded in a decolonial framework, the project centers Māori authority, self-determination, and collective well-being, and aims to inform policy and challenge restrictive legislation. Findings from this feasibility study will inform a subsequent Phase-2 patient trial. The initiative generates both formal clinical data and Indigenous-defined endpoints, expanding conventional models of efficacy and safety in psychedelic research. The Māori Tū Wairua project currently represents the clearest example of an Indigenous-governed psilocybin intervention consistent with United Nations Declaration on the Rights of Indigenous Peoples (UNDRIP) principles (a universal framework of minimum standards for the survival, dignity, and well-being of Indigenous peoples worldwide), in that the protocol is led by Māori authorities, conducted on Māori terms and land, and operationalizes Indigenous-defined endpoints and benefit structures.

A different model of adaptation is proposed in Mexico, where researchers are exploring how Indigenous psilocybin practices can be translated into a regulated biopsychosocial framework without severing their cultural, pharmacological, or ecological grounding. Escamilla et al. [75] propose an Indigenous-informed psilocybin-assisted therapy program for Major Depressive Disorder in Mexico, integrating national biocultural resources into a staged translational model. The working group preferred naturally derived *Psilocybe cubensis* over synthetic psilocybin for reasons of cultural continuity, local cultivability and regulatory sovereignty. The three-stage design includes (i) chemical and preclinical characterization, (ii) safety testing in healthy volunteers, and (iii) an open-label patient trial comparing mushroom-assisted psychotherapy with standard care. A distinctive feature is the explicit incorporation of Indigenous practice elements, whole-mushroom use, group format, ritual specialists, culturally grounded illness models, and integrative care. The authors argue that such a program could demonstrate that naturally derived psilocybin can be standardized and ethically adapted without stripping it of its culturally embedded therapeutic logic. This study incorporates Indigenous knowledge and context, but is not Indigenous-governed.

#### 3.2.3. Experimental Studies in Naturalistic and Semi-Naturalistic Contexts

##### Uncontrolled Open-Label Studies with Microdosing

Early open-label field studies suggested that psilocybin microdosing may enhance creativity and empathy. Kuypers et al. [76] and Prochazkova et al. [77] both conducted research in naturalistic settings during community-based events organized by psychedelic interest groups and reported improvements in divergent and convergent thinking after psilocybin truffle microdosing, while fluid intelligence remained unaffected. Mason et al. [78] in a retreat setting, found sub-acute increases in divergent thinking, empathy, and well-being the morning after psilocybin intake, with gains in convergent thinking, empathy, and well-being persisting one week later. Together, these studies suggest possible benefits for creativity and social–emotional processes, but their open-label, uncontrolled designs limit firm conclusions.

##### Controlled Microdosing Studies

A number of studies have compared microdosing to various forms of placebo. Szigeti et al. [79] conducted an innovative randomized, placebo-controlled citizen science trial using a self-blinding design with 191 participants (57 using psilocybin). Over four weeks, both microdosing and placebo groups showed improvements in psychological well-being, but no significant between-group differences emerged. Small advantages for microdosing appeared in acute mood, energy, and creativity, as well as post-acute reductions in anxiety, though the authors highlighted the role of expectancy and unblinding.

Cavanna et al. [80] conducted a double-blind, placebo-controlled study of 34 novices microdosing psilocybin. Participants under psilocybin reported stronger acute subjective effects and showed EEG changes (reduced theta power), but no improvements in creativity, cognition, or well-being were observed compared to placebo. Minor signs of cognitive impairment emerged, and the authors concluded that expectation likely accounts for much of the anecdotal benefit.

Marschall et al. [81] conducted a double-blind, placebo-controlled crossover trial, investigating the effects of a three-week psilocybin microdosing regimen on emotion processing and mental health symptoms. They found that microdosing did not significantly affect emotion processing, self-reported interoceptive awareness, or symptoms of anxiety and depression compared to the placebo condition.

Prochazkova et al. [82] reported two double-blind studies investigating the impact of a single psilocybin microdose on cognitive control, memory, social cognition, and well-being. The findings indicated that microdosing did not reliably enhance cognitive performance or emotional functioning beyond the effects of a placebo. In a separate analysis, Prochazkova et al. [83] pooled data from three double-blind, placebo-controlled trials (total N = 171) to explore microdosing’s effect on divergent and convergent thinking. The study found that active microdosing significantly increased the originality-to-fluency ratio, which suggests an enhancement in the quality and novelty of divergent thinking. However, microdosing had no significant effect on convergent thinking or other divergent metrics like raw fluency or flexibility.

van Elk et al. [62] conducted a preregistered study using a double-blind, placebo-controlled crossover design to evaluate the effects of psilocybin microdosing on feelings of awe and esthetic experiences. Confirmatory analyses showed that participants reported increased feelings of awe in response to neutral and positive videos while microdosing compared to the placebo condition.

Enriquez-Geppert et al. [84] conducted a randomized semi-naturalistic feasibility study using psilocybin-assisted neurofeedback to enhance frontal–midline theta, a neural marker of executive functioning. The study hypothesized that psilocybin would boost neural plasticity and neurofeedback responsiveness. A total of 37 participants were randomized to an experimental group (n = 18; a week of microdosing alone, followed by three psilocybin-assisted neurofeedback sessions) and a passive control (n = 19). Neurofeedback learning showed a session-by-session theta increase trend (large effect size), but no improvements in within-session theta changes or task-based executive functions. However, participants reported significant self-perceived improvements in executive functions (working memory, shifting, monitoring, inhibition; medium–large effect sizes) and often achieved self-defined goals (cognition, presence, mood). The intervention, using psilocybin truffles, was safe, tolerable, and feasible, with no dropouts or adverse psychological effects.

Overall, controlled microdosing studies have not shown strong evidence for cognitive or clinical benefits of psilocybin microdosing. However, there are a number of important factors that limit the implications that can be drawn from these studies. First, a significant challenge across all studies was the failure of the blinding procedure, as participants often correctly identified their condition based on subtle physiological side effects like nausea, increased heart rate, and sweating. Second, these studies mostly investigated the effects of a single or a small number of doses, and may not have provided enough exposure to generate the effects reported by microdosers in real-world settings. Third, these studies have mainly used extremely low doses of psilocybin that may be too small even for a microdose effect (see [63] for a detailed evaluation of controlled studies of microdosing).

### 3.3. Criterion 2: Safety and Tolerability

This criterion evaluates whether there is documented evidence that naturally derived psilocybin can be used safely in human populations and under what conditions tolerability may be compromised. Here, safety refers to the likelihood and severity of acute or delayed adverse physiological or psychological effects, tolerability to an individual’s capacity to endure the subjective and somatic effects of psilocybin without significant functional disruption or clinical deterioration.

Psilocybin has very low toxicity, is non addictive, and there is only one known case of overdose [85]. There are, however, important risks and side effects. Psilocybin causes transient alterations to perception and cognition, which can lead to dangerous behaviors. Psilocybin is also associated with a number of psychiatric side effects, including anxiety, paranoia, derealization, depersonalization and hallucinogen persisting perception disorder [86].

Most current evidence on the safety profile of naturally derived psilocybin is observational. This section is organized thematically, covering adverse effects, pharmacological interactions, emergency care, and practitioner insights.

#### 3.3.1. Defining Adverse Events Across Clinical and Cultural Contexts

The concept of “adverse events” is not universally fixed but deeply influenced by setting, expectation, and cultural framing. For instance, in clinical research with synthetic psilocybin, adverse events are typically defined as undesirable symptoms or complications that require medical intervention, prolonged hospitalization, or compromised physical or psychological functioning [87]. According to safety guidelines such as those by Johnson et al. [88], symptoms like nausea, vomiting, or anxiety are coded as adverse events and often used as indicators of poor tolerability. However, in Indigenous or ceremonial contexts, the same phenomena may be interpreted very differently. For example, vomiting or diarrhea during ayahuasca or psilocybin rituals is often regarded as a purging or cleansing process, a necessary step toward healing rather than a side effect to be minimized [72,89]. These interpretations are not only symbolic; they are increasingly supported by research into the gut–brain axis, suggesting that certain physiological responses may in fact contribute to the therapeutic effect [90,91]. Similarly, strong visions, dissociative states, or emotional overwhelm are often seen as problematic in clinical settings, particularly when linked to acute distress or diagnostic instability. Yet within naturalistic and traditional use frameworks, such intense experiences are often considered therapeutically meaningful, enabling the resolution of emotional blockages, trauma processing, or spiritual insight.

These differences raise important questions about how adverse events should be interpreted in the evaluation of naturally derived psilocybin use. Although these side effects are often clear indications of potential risks, they also underscore the need for context-sensitive safety frameworks that recognize the value of cultural meaning-making, subjective interpretation, and the goals of the user.

#### 3.3.2. Adverse Effects and Psychological Challenges

##### Survey-Based Evidence

Survey-based evidence on adverse effects associated with psilocybin use is primarily derived from the GDS datasets. However, some studies combine psilocybin with other substances (e.g., LSD), limiting possibly in specific conclusions.

In the study by Kopra et al. [50] on GDS 2020 data on self-treatment with LSD and psilocybin mushrooms, 23% (n = 705) of respondents reported negative effects, most commonly mental confusion, memory issues, or a sense of disconnection. Onset typically occurred within 24 h (~60%), though 20% noticed effects after one week. Most adverse effects resolved within 7 days (~55%), though 27% lasted a month or more. A total of 4.2% sought emergency care once, and 0.5% sought care more than once.

Data from GDS 2021 [34] showed that 77.6% of psilocybin users reported no adverse effects in the prior 12 months. Among those who did, adverse effects were categorized as mental (8.3%), physical (6.4%), tolerance to desired mental effects (3.4%), tolerance to desired physical effects (1.5%), and other undesired effects (2.7%). Less than 1% of psilocybin users sought emergency medical treatment.

Analysis of the GDS 2019 on microdosing [58] revealed that when difficulties were noted among psilocybin microdosers, they most often involved physiological discomfort, impaired energy (restlessness/fatigue), or cognitive interference (e.g., confusion, memory issues, racing thoughts).

Using GDS 2017 data (*n* = 9233), Kopra et al. [92] found that only 0.2% of past-year psilocybin mushroom users (n = 19) sought emergency medical treatment, with a per-event risk of 0.06%. Incidents were most often characterized by anxiety, panic, or paranoia, and were commonly linked to poor set or setting and substance mixing. Young age was the only significant predictor of risk. All but one case resolved within 24 h, underscoring that while acute adverse reactions can occur, they are rare, often short-lived, and typically non-severe.

##### Qualitative Evidence from Social Media: Self-Reported Negative Outcomes

Bienemann et al. [93] analyzed 346 social media reports using quantitative textual analysis to identify recurring themes in self-reported negative outcomes of psilocybin use. The study revealed four key clusters: thinking distortions, medical emergencies, perceptual alterations, and substance administration. Findings showed that “bad trips” were more frequent in female users and strongly linked to thinking distortions, long-term negative outcomes correlated with multiple doses in one session or polysubstance use, and single high doses increased the risk of medical emergencies. The associations between bad trips and gender, as well as polysubstance use and long-term outcomes, generate hypotheses for further research, particularly regarding sex differences in substance response and the risks of drug interactions. However, findings should be interpreted cautiously due to potential self-selection bias and the non-representative nature of online reports.

#### 3.3.3. Pharmacological Interactions

##### Large-Scale International Online Surveys

Lake and Lucas [35] analyzed GDS 2023 data (*n* = 5370 psychedelic users, including 4955 psilocybin users) to assess co-use patterns with 11 psychoactive substances. Most psilocybin users (66%) reported no co-use, while the most common combinations were with cannabis and alcohol. Depressant co-use was uncommon (<20%), though some increase was noted post-psychedelic experience. Co-use was generally associated with stronger psychedelic effects and recreational or substitution motives, but was less frequent among those using psilocybin for personal exploration or therapeutic purposes. While most co-use appeared limited to cannabis and alcohol, the practice nonetheless introduces potential risks due to unpredictable drug interactions.

##### Survey and Qualitative Research on Polysubstance Use

Zeifman et al. [94] examined whether combining 3,4-Methylenedioxymethamphetamine (MDMA) with psilocybin (or LSD) alters subjective experiences in an online survey (n = 698; 356 psilocybin, 342 LSD, 27 co-users). Compared with psilocybin or LSD alone, low-dose MDMA co-use was linked to fewer challenging experiences (including grief and fear) and greater positive emotions such as self-compassion, love, and gratitude. Combining psilocybin with MDMA did not affect mystical-type experiences or compassion ratings. These findings suggest that adding low-dose MDMA may buffer against difficult psilocybin experiences while enhancing certain affective outcomes, though evidence remains limited by the small number of co-users, the non-experimental design and the nature of self-reports.

Licht et al. [95] combined structured interviews and hair analysis of psilocybin and LSD users to examine polysubstance use (*n* = 98). Co-use was widespread: cannabis (81% psilocybin; 78% LSD) and alcohol (64%; 79%) were most common, followed by MDMA (31%; 52%). Participants described synergistic effects (e.g., cannabis or MDMA enhancing hallucinogen effects) as well as counteracting effects (e.g., stimulants or cocaine dampening hallucinogens). Polysubstance use was more frequent with LSD than with psilocybin, but the majority of both groups reported often or always co-using alcohol and cannabis.

##### Interactions with Pharmaceutical Medications

A systematic review by Halman et al. [96] examining drug interactions with classic psychedelics, including naturally derived psilocybin, reported that SSRIs and monoamine oxidase inhibitors (MAOIs) may attenuate subjective psychedelic effects, whereas tricyclic antidepressants may enhance them. Similarly, Gukasyan et al. [97] surveyed 611 individuals combining psilocybin mushrooms with SSRIs or SNRIs. Approximately half reported attenuated acute effects, with no clear dose–response relationship. Consistent with this, Sakai et al. [98] analyzed Reddit posts on SSRI and psilocybin co-use, finding that 54% described reduced intensity, 39% no change, and 8% negative effects such as headache, nausea, confusion, or possible serotonin toxicity or manic/psychotic symptoms. These findings suggest that SSRI/SNRI co-use may reduce subjective effects, though safety conclusions remain uncertain due to self-report and selection biases. Together, these findings suggest that SSRI/SNRI co-use often dampens psilocybin effects but may also introduce safety risks, underscoring the need for caution and systematic study of drug–drug interactions.

Interactions with opioid-related pharmacotherapies used in addiction rehabilitation, such as naloxone and methadone, have not been directly studied. Psilocybin’s serotonergic mechanism (5-HT_2A_ agonism via psilocin) is pharmacologically distinct from the opioid-receptor activity of these agents, and no direct interaction has been documented. However, methadone’s weak serotonergic activity and its association with QT-interval prolongation [99] warrant caution, and shared hepatic metabolic pathways represent a plausible but unstudied site of interaction.

Evidence of serious adverse interactions is limited to isolated case reports. Barnett et al. [100] described a hypertensive emergency and myocardial infarction in a patient combining psilocybin mushrooms with tranylcypromine (an MAOI) and amphetamine salts. While causality cannot be established from a single case, it highlights a potential safety signal, particularly involving MAOIs or stimulants, and underscores the importance of medication screening and clinical supervision.

#### 3.3.4. Natural–Psilocybin Specific Risks and Contamination-Related Syndromes: Ethnographic Analysis, Survey Studies

Survey evidence has identified a distinct toxidrome known as wood-lover paralysis (WLP), associated with ingestion of lignicolous (“wood-loving”) *Psilocybe* species. Beck et al. [101] surveyed 392 individuals and found that symptoms typically began within four hours and included muscle weakness lasting up to three days. In severe cases, weakness impaired movement, chewing, or swallowing, posing risks such as falls, choking, or breathing difficulties. WLP has been reported almost exclusively after wild-harvested mushrooms, suggesting environmental or substrate-related causes and indicating that controlled cultivation and standardized production may substantially reduce this risk.

Misidentification represents another significant safety concern, as psilocybin-containing mushrooms can resemble highly toxic species [102]. The Oregon Psilocybin Advisory Board [103] therefore recommends expert identification methods, including DNA-based verification. Even when correctly identified, natural mushrooms exhibit substantial variability in alkaloid content, with psilocybin concentrations ranging from approximately 0.01% to 2% of dried weight, contributing to unpredictable dosing and effects. Ongoing observational research is examining how species and strain variability influence subjective and physiological outcomes [104].

Ethnographic evidence provides additional contextual safety insights. Haro-Luna et al. [105], in an ethnographic study of Zapotec ceremonial use, noted risks related to misidentification and adverse psychological or physical effects, and described traditional harm-reduction practices such as supervised consumption and avoidance of other substances.

#### 3.3.5. Facilitator Perspectives on Safe and Effective Psilocybin Use

Hughes et al. [39] interviewed 17 underground psilocybin facilitators in the Western US, most of whom were white, female, and experienced, often with years of personal psilocybin use before guiding others. Practitioners stressed safety concerns, particularly for clients with trauma, personality disorders, or little social support, and warned against inadequate preparation or rapid escalation to high-dose sessions.

Wilson-Poe et al. [106], through qualitative interviews with 36 experienced psilocybin facilitators (mean 15 years’ experience), highlighted that personal psychedelic use was widely seen as a key qualification for providing safe and effective care, though not unanimously endorsed. Such first-hand experience was viewed as enhancing facilitators’ well-being, empathy, and ability to contextualize clients’ experiences. These qualitative findings provide a valuable perspective from practitioners working directly with psilocybin users, often in non-clinical contexts. Their insights highlight the nuanced psychosocial dimensions of safety, the role of lived experience in facilitation, and the need for careful, trauma-informed practice. This study underscores the potential value of experiential knowledge in informing clinical models of psychedelic care.

#### 3.3.6. Single Case Studies Reporting Adverse Events

Case reports illustrate severe adverse outcomes associated with psilocybin use: Kim et al. [107] described three cases of acute psychosis (prolonged symptoms in bipolar disorder, severe suicidality, and aggression) with variable treatment responses; Morris [108] documented psilocybin-induced psychosis with catatonia and suicidality in a vulnerable patient (depression, personality traits, cannabis use) after heavy use, reinforcing that adverse reactions predominantly occur in high-risk individuals using excessive doses. Perna et al. [109] reported prolonged adverse effects (anhedonia, sleep impairment, and suicidal ideation) in a psychologist after repeated high-dose psilocybin in an unregulated training program, requiring ECT for resolution. Finally, a fatal case of self-inflicted trans-orbital trauma (penetrating brainstem injury) following psilocybin ingestion [110] highlights extreme risks of acute behavioral dysregulation. While these individual reports suggest potential dangers, particularly in vulnerable populations or unsupervised settings, they do not establish causality or prevalence, emphasizing the urgent need for systematic studies to clarify risks, safety protocols, and contraindications in both recreational and therapeutic contexts.

#### 3.3.7. Dose-Dependent Physiological Safety: Insights from Synthetic Psilocybin Studies

Animal studies suggest that psilocybin exhibits dose-dependent toxicity at sufficiently high concentrations. High-dose psilocybin (10 mg/kg) produced oxidative damage in rat hippocampal and frontal brain regions, whereas lower doses (2 mg/kg) did not, suggesting a potential dose-dependent neurotoxicity threshold [111]. Notably, even this lower dosage substantially exceeds those typically administered in human research.

In humans, the dose required to produce obvious psychological effects has been estimated to be around 10 mg [112]. Controlled clinical studies further demonstrate dose-dependent physiological responses and subjective intensity ratings. For example, Straumann et al. [113] compared doses of 15 mg, 20 mg, 25 mg and 30 mg in 85 healthy participants and found that 15 mg produced weaker subjective effects, compared to the other dosages. Additionally, body temperature was shown to increase with the dosage. Adverse effects, such as increased anxiety, occurred only in the 25 and 30 mg doses. Similarly, a double-blind study comparing 15 mg and 30 mg doses found dose-dependent increases in subjective effects, blood pressure, heart rate, and body temperature, which remained within tolerable safety limits [114].

Neuroimaging further supports a dose-dependent relationship. Madsen et al. [115] found a dose-dependent change in the cerebral 5-HT_2A_ receptor (5-HT_2A_R) binding in eight participants. Not only were the psilocin levels (3–30 mg) correlated with the 5-HT_2A_R occupancy, but an additional correlation was found with the intensity ratings of the participants. This suggests clinical implications for dose-dependence on psychedelic experience. Although these findings cannot fully account for variability in naturally derived psilocybin, they provide important indirect reference points for dose-related responses and physiological safety.

### 3.4. Criteria 3: Unique Psychopharmacological Profile (Vs. Synthetic)

This section examines the physiological and psychological differences between naturally derived and synthetic psilocybin. It begins with a chemical overview of psilocybin and potential entourage compounds, followed by a review of computational models, preclinical research and human evidence.

#### 3.4.1. Brief Chemical Overview of Psilocybin and Possible Entourage Compounds

Psilocybin and its structurally related alkaloids baeocystin, norbaeocystin and aeruginascin are tryptamines, sharing a common indole core. A brief overview of these compounds is provided in Table 2.

Recently, Fricke et al. [116] identified key fungal enzymes and reconstituted the biosynthetic pathway. The biosynthesis begins with the amino acid tryptophan being decarboxylated to afford tryptamine. In the next step, a hydroxy group is attached to the tryptamine by a common P450 monooxygenase. The respective product is Norbaeocystin. Successive methylations produce baeocystin, baeocystin and psilocybin as homologues. Aeruginascin’s biosynthesis remains unclear. While this metabolite is common in many producers, including transgenic yeast expressing the four psilocybin biosynthesis genes, PsiM’s role in the third methylation is unconfirmed.

**Figure 1:**
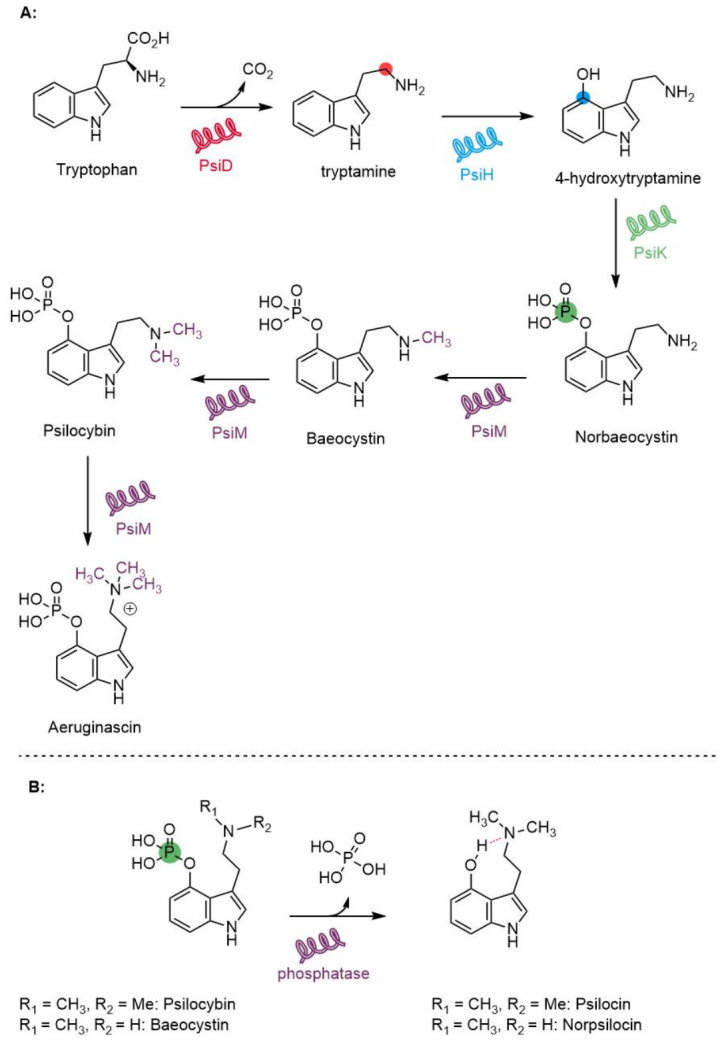
Biotransformations of psilocybin alkaloids. (**A**) Biosynthesis; (**B**) phosphate cleavage yielding the bioactive compounds.

Phosphorylated compounds (norbaeocystin, baeocystin and psilocybin) are not able to passively cross the blood–brain barrier (BBB). After ingestion, the digestive enzymes cleave the phosphate group. This is relevant for baeocystin and psilocybin, which, by this mechanism, liberate psilocin and norpsilocin, which can passively pass the BBB. An intriguing structural feature of psilocin, which has been a long-standing debate [117], is the interaction between hydrogen and nitrogen (Figure 1B) via a hydrogen bond (red dotted line) and has two key effects. First, this hydrogen bond enhances the lipophilicity of psilocin, increasing BBB penetration and affects the central nervous system. Second, this hydrogen bond attenuates the MAO degradation of psilocin. Table 3 summarizes the biological role and effects of psilocin.

Psilocin binds primarily to serotonin receptors (5-HTRs). It has weak affinity for monoamine transporters and other monoamine receptors, see Table 3. Affinities vary by studies [118] due to experimental conditions. Factors like tissue type, cell type, receptor density, and cell media all affect the measured binding affinities, but relative affinities remain informative.

5-HT_1A_ receptors (5-HT_1A_Rs), located primarily presynaptically (raphe serotoninergic nuclei) and postsynaptically (cortical and limbic regions), modulate psychedelic and psychoplastogenic effects of psilocin [119,120,121,122]. Psilocin core effects, psychedelic and psychoplastogenic, are primarily mediated through partial agonism at the 5-HT_2A_R. 5-HT_2A_Rs are located presynaptically on axon terminals and postsynaptically on somatodendritic or intracellular compartments. It is the most abundant excitatory 5-HT receptor. Layer 5 pyramidal cells and GABAergic interneurons in the medial prefrontal cortex (mPFC) have high 5-HT_2A_R density [123,124,125]. Psilocin also binds to the 5-HT_2C_ receptor (5-HT_2C_R), which has been implicated in the modulation of dopaminergic signaling in the ventral tegmental area (VTA), involved in impulse regulation [123].

##### Downstream Signaling: Impacts on Neuroplasticity and Functional Neural Networks

Psilocin activates both 5-HT_2A_-G_q/11_ and β-arrestin2 transducers through binding to the 5-HT_2A_R in two distinct modes, a β-arrestin2 biased mode and a G_q/11_ signaling biased mode. The G_q/11_-mediated signalling cascade results in increased excitatory glutamatergic neurotransmission and receptor internalization. The β-Arrestin2 pathway leads to distinct internalization and downstream signalling cascades, including extracellular signal-regulated kinase (ERK) activation, which is involved in synaptic plasticity. β-Arrestin2-biased 5-HT_2A_R agonists fail to produce the head twitch response (HTR) in mice, which is a predictor of psychedelic potential. HTR requires threshold G_q/11_ efficacy [121,125,126,127]. β-Arrestin2-biased low G_q/11_-E_MAX_ 5-HT_2A_R agonists act as non-psychedelic psychoplastogens. These biased partial agonists are under investigation as novel treatments for neuropsychiatric diseases (depression and schizophrenia) linked to cortical dendritic spine loss [125,128,129].

The psychoplastogenic effect is sustained for an extended period after psilocin has been cleared from the extracellular space. A portion of the cortical 5-HT_2A_Rs is localized in the Golgi, which is slightly more acidic than the cytosol and extracellular space. Psilocin can permeate through the cellular membranes and enter the Golgi, where the acidic environment allows for protonation of psilocin’s basic nitrogen. This effectively traps psilocin within the Golgi, which allows for sustained intracellular 5-HT_2A_R signaling and may be the cause of the sustained psychoplastogenic effect. Intracellular 5-HT_2A_Rs may also contribute to the psychedelic effect, as serotonin-releasing agents can produce the HTR in mice expressing the serotonin transporter (SERT) on cortical neurons of the mPFC but not wild-type mice [130].

Layer 5 pyramidal cells in the mPFC, rich in 5-HT_2A_Rs, are highly sensitive to psilocin. Their activation induces a glutamate-dependent increase in the activity of pyramidal neurons. Glutamate stimulates post-synaptic α-amino-3-hydroxy-5-methyl-4-isoxazolepropionic acid receptors (AMPARs) and N-methyl-D-aspartate receptors (NMDARs), leading to initiation of signaling cascades which promote dendritic spine enlargement and the formation of new spines (spinogenesis) through upregulation of brain-derived neurotrophic factor (BDNF), mammalian target of rapamycin (mTOR), eukaryotic elongation factor 2 (eEF2), and other related genes [119,121,122,131,132]. Both 5-HT_2A_R and postsynaptic 5-HT_1A_R-mediated signalling cascades also contribute to psychoplastogenic effects in the hippocampus. The psychoplastogenic effect of psilocin is larger in the mPFC than in the hippocampus, as psilocin has a greater affinity for 5-HT_2A_Rs than for 5-HT_1A_Rs [119,122,133]. The granular retrosplenial cortex (GRC) lacks 5-HT_2A_Rs, but presynaptic 5-HT_2A_Rs activation on anterior thalamic axonal inputs to the GRC has been shown to increase synaptic connectivity in the GRC [124].

Psilocin has also been shown to act as an allosteric modulator of tropomyosin receptor kinase B (TrkB), which physiologically binds neurotrophins such as BDNF. Deficiencies in BDNF signaling through TrkB have been implicated in the pathophysiology of depression and neurodegenerative disorders such as Alzheimer’s disease, Parkinson’s disease, and Huntington’s disease, which has led to an increase in interest in TrkB as a target for drug development [134]. By binding to the transmembrane domain of the TrkB dimer, psilocin maintains a receptor conformation that is optimal for receptor activation [134,135]. However, this discovery has been challenged by another study that failed to replicate the results [136].

Electrophysiological studies offer another perspective on the changes that occur within the brain under the influence of psilocin. One of the primary observed effects is pronounced disruption of functional connectivity (FC) in the cortex and subcortex, with changes being most significant in the default mode network (DMN) [131]. The DMN consists of a distributed, interconnected set of distinct brain regions that are functionally and spatially distant from the peripheral brain systems. It is involved in abstract cognition, often unrelated to the immediate environment. Neuronal activity within the DMN is decreased during complex, attention-demanding tasks [137].

Beyond the psychedelic and psychoplastogenic effects, psilocin is also believed to influence senescence. As a large number of studies link adverse psychological conditions to telomere attrition, Germann [138] hypothesized that psilocybin may have a quantifiable impact on their length. To test the hypothesis, Kato et al. treated fetal lung fibroblasts with psilocin and found a dose-dependent decrease in markers of cell cycle arrest, increases in markers of proliferation, and extensions in cellular lifespan of up to 57%. Telomere length was preserved in psilocin-treated cells while vehicle-treated cells exhibited reduced telomere length. The same study treated aged mice with psilocybin and found significantly higher survival (80%) when compared to vehicle (50%) [138,139].

#### 3.4.2. Mechanisms of the Entourage Effect

The entourage effect, a phenomenon wherein co-occurring bioactive compounds modulate the primary psychoactive effects of a substance, has been characterized in cannabinoids [140,141] and has emerged as a key consideration in psilocybin research. In psilocybin-containing mushrooms, secondary compounds (e.g., baeocystin, norbaeocystin, norpsilocin, aeruginascin, β-carbolines) are absent in synthetic psilocybin but may enhance, prolong, or alter therapeutic and psychoactive effects through pharmacodynamic and pharmacokinetic interactions. Proposed mechanisms of the entourage effect include pharmacodynamic interactions and/or pharmacokinetic interactions. Pharmacodynamic interactions would involve direct modulation of receptor activity in the central nervous system. Pharmacokinetic interactions could occur earlier, including altered absorption, metabolism, or elimination. For example, β-carbolines identified in psilocybin mushrooms have monoamine oxidase (MAO) inhibitory properties and could theoretically influence psilocin metabolism, although detected concentrations appear too low to produce clinically meaningful effects [142]. If natural forms of psilocybin have different effects from synthetic psilocybin, then it must be because these entourage compounds (or other currently undiscovered entourage molecules) have independent psychoactive effects, or because they somehow potentiate or otherwise change the effects of psilocybin.

#### 3.4.3. Computational Models

A computational study by Murray et al. [143] investigated the entourage effect of naturally derived psilocybin by comparing the neuropharmacological mechanisms of unpurified mushroom extracts to those of *isolated psilocybin* (i.e., to the psilocybin molecule on its own). Using network pharmacology, molecular docking, and molecular dynamics simulations, the authors identified eight bioactive compounds (e.g., psilocin, harmaline, harmane, harmole, 4-Hydroxytryptamine, 4-Hydroxy-N,N,N-trimethyltryptamine, Phenylethylamine) with favorable BBB permeability and low toxicity. These compounds interact with 44 brain-localized proteins, primarily within serotonergic (HTR_2A_), dopaminergic (DRD2), and monoamine oxidase (MAOA) pathways, suggesting synergistic modulation of neuropsychiatric targets. Molecular docking revealed strong binding interactions (e.g., salt bridges with Asp155 in HTR_2A_), while 200 ns MD simulations confirmed stability, particularly for β-carboline-MAOA complexes, implying potential MAOA inhibition. Functional enrichment highlighted roles in neurotransmission, synaptic plasticity, and cardiovascular regulation. The study provides a mechanistic rationale for the potential enhanced efficacy of naturally derived psilocybin over pure isolated psilocybin, emphasizing the need for further preclinical and clinical validation.

#### 3.4.4. Preclinical Research

Preclinical behavioral studies show mixed results. Zhuk et al. [144] reported that synthetic psilocin induced greater HTR compared to naturally derived psilocybin extracts. In contrast, Shahar et al. [145] found similar HRT responses between natural and synthetic compounds. Notably, Shahar et al. found preliminary indications that naturally derived psilocybin may produce a more potent and prolonged effect on synaptic plasticity than synthetic psilocybin, shown by: (a) increased expression in four synaptic proteins across the frontal cortex, hippocampus, amygdala and striatum, (b) metabolic analysis revealing broader biochemical changes compared to synthetic psilocybin, though plasma psilocin levels remained unchanged. Although preliminary, this is an indication that naturally derived psilocybin may induce more robust and sustained neuroplastic changes.

Two studies assessed behavioral and neurochemical effects of individual compounds. Sherwood et al. [146] demonstrated that baeocystin on its own did not activate the 5-HT_2A_R in mice. Rakoczy et al. [147] identified norbaeocystin as a potential contributor to antidepressant-like effects in rats in the forced swimming test. Unlike psilocybin, norbaeocystin did not induce HTR, suggesting a distinct mechanism of action potentially involving 5-HT_2A_R activation without hallucinogenic effects.

Matsushima et al. [148] investigated the effects of naturally derived psilocybin on marble-burying behavior in mice, a model of OCD-like behavior. They found that naturally derived psilocybin reduced marble-burying behavior at lower doses than synthetic psilocybin. Neither substance adversely affected locomotor activity, suggesting a specific anxiolytic/OCD-like effect. HPLC quantification confirmed the presence of psilocybin, but the enhanced efficacy was attributed to additional bioactive compounds.

These studies provide preliminary indications that naturally derived psilocybin may have increased therapeutic benefits relative to synthetic psilocybin, possibly due to the activity of norbaeocystin, however confirmatory studies and in vivo validation of computational predictions are needed.

#### 3.4.5. Human Evidence

Despite preclinical promise and frequent claims, human research on the entourage effect remains sparse and largely theoretical. Many assertions regarding baeocystin, aeruginascin, or norbaeocystin derive from anecdotal reports [149], case descriptions, or secondary citation chains with minimal primary experimental validation. Available pharmacological analyses suggest that some compounds, such as aeruginascin, may have limited brain penetrance or reduced receptor activity compared to psilocin [150], although in vivo human data are lacking. However, user preferences and cultural perceptions strongly favor natural sources.

##### Large-Scale International Surveys on Preferences for Naturally Derived vs. Synthetic Psilocybin

Syed et al. [151], using GPS 2023 data (*n* = 6379), examined preferences and perceptions regarding natural and synthetic psychedelics. A strong preference for natural sources was reported, particularly for psilocybin (75%), as well as for DMT (56%) and mescaline (56%). About half of respondents (50.8%) believed that the source of the psychedelic influences its psychological and physiological effects, while one-third (34.4%) expressed a neutral stance. Similar attitudes are evident in the Māori-led Tū Wairua Project, which plans to administer naturally derived psilocybin in a trial targeting problematic methamphetamine use. Naturally derived psilocybin is preferred by this community as it is believed to contain greater healing properties than synthetic forms [74]. These findings highlight that beyond motives for use, perceptions of *naturalness* itself may shape users’ attitudes and choices regarding psilocybin. These preferences may independently shape the subjective effects and outcomes that users experience.

##### Historical and Contemporary Reports on Subjective Effects

Specific reports on the phenomenology of synthetic compared to naturally derived psilocybin give some indications of the subjective effects of these substances. The historical psychedelic literature provides several notable accounts of subjective effects. Albert Hofmann was the first person to synthesize psilocybin, and he included an account of his own self-assay of the synthetic form in his book *LSD: My problem child* [152]. He describes a strongly hallucinogenic and frightening experience: “*For me, this entry into the mushroom world had been a test, a confrontation with a dead world and with the void*”. He did not make direct comparisons with the subjective effects of naturally derived psilocybin but did contrast the experience with LSD, saying: “*Personally, for me, there was more light in the LSD experiments than in the experiments with the earthy mushroom*”. Intriguingly, Hofmann also included a report on the effects of synthetic psilocybin from Maria Sabina, the Mazatec curandera who first shared psilocybin containing mushrooms with westerners. Sabina, although originally skeptical due to the slower onset, ultimately judged the synthetic pills to have the same effects as organic mushrooms:

*“As we took leave of María Sabina and her clan at the crack of dawn, the curandera said that the pills had the same power as the mushrooms, that there was no difference. This was a confirmation from the most competent authority that the synthetic psilocybin is identical with the natural product. As a parting gift, I let María Sabina have a vial of psilocybin pills. She radiantly explained to our interpreter, Herlinda, that she could now give consultations even in the season when no mushrooms grow”.* p. 152.

In one of the early scientific reports of the effects of synthetic psilocybin, Pahnke [153] reported intense changes in conscious awareness characterized by intense feelings of unity and transcendence that mapped the features of traditional mystical experiences. Smith [154] similarly reported a self-experiment by psychologist Wilson Van Dusen, who likened the experience to an advanced state of Zen meditation. Broadly, reports from the early clinical and scientific use of synthetic psilocybin contain similar accounts of boundary dissolution and mystical themes to reports describing naturalistic use of psilocybin containing mushrooms.

##### Empirical Human Research

So far, there has been only a single study directly comparing the effects of different forms of psilocybin in humans. In a qualitative study, Kryskow et al. [155] administered four patients various doses of synthetic psilocybin, whole *Psilocybe cubensis* mushrooms or unpurified mushroom extract. Three of these patients experienced both the synthetic and a naturally derived form of psilocybin (either whole mushrooms or the unpurified mushroom extract). Participants reported broadly similar clinical, psychological and subjective effects for the three psilocybin forms, however there were some nuanced points of difference. For example, synthetic psilocybin was described as less colorful, more “mechanical,” and slightly less emotional and euphoric than other forms. Participants also reported synthetic psilocybin to be less spiritual and sacred than whole mushrooms or unpurified mushroom extract forms. Participants did express a clear preference for whole mushrooms, describing this form as more natural and conferring a greater overall quality of experience. However, this was an open-label study, and it is possible that participants’ judgements regarding the quality of psilocybin mushrooms were influenced by beliefs and expectations. Notably, the treatment in this program was presented in a ceremonial context, which may have primed preferences for natural substances.

### 3.5. Criterion 4: Identity and Composition Control

This criterion deals with the complexity of *Psilocybe* species as a source of psychoactive alkaloids. First, an overview of the chemical composition of various species is provided. This is followed by the most common processing of the natural sources and a brief discussion of the implications for the composition control. The last section deals with the chemical stability of psilocin and psilocybin in the natural sources and during their processing.

The exact number of psilocin and psilocybin producing mushrooms is not precisely known, but according to Guzmán [156], there are at least 144 hallucinogenic species. These are distributed over all continents, with Mexico and Latin America (including the Caribbean) providing conditions for growth to more than 53 and 50 species, respectively. The US and Canada have 22 species, Australia has 19, Europe has 16, Asia has 15, and Africa has four species. Some *Psilocybe* species are common throughout several regions. For example, *P. cubensis* and *P. subcubensi* are found in all tropical regions, and *P. argentina* is found in several high mountains and the Northern and Southern Hemispheres. Furthermore, *P. fimetaria* and *P. semilanceata* are known in Europe, Canada, and the US; however, not in Mexico.

**Figure 2:**
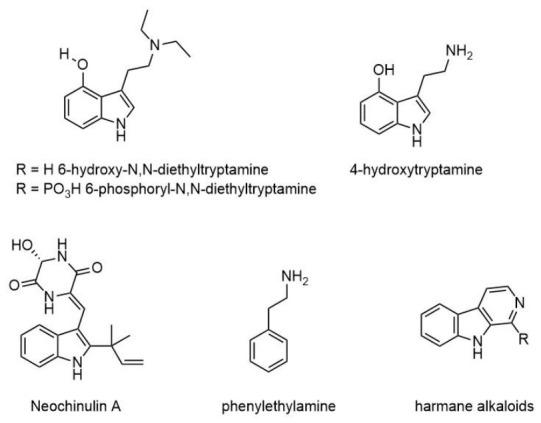
Chemical names and structures of relevant alkaloids and other psychoactive compounds found in *Psilocybe* mushrooms.

There are numerous natural products available from *Psilocybe* mushrooms, including alkaloids, terpenoids and also other hard-to-classify compounds. In this overview, we limit the possible compounds to homologues of psilocin and psilocybin (see below, Figure 2), such as 6-hydroxy-N,N-diethyltryptamine (and phosphorylated analog) and 4-hydroxytryptamine. Furthermore, those that show neuroprotective and anti-oxidant properties, Neochinulin A and compounds such as phenylethylamine and various harmanes, which are known psychoactive compounds. A more detailed overview of the chemical composition of *Psilocybe* mushrooms can be found in an excellent review by Luz et al. [157].

In general, the literature does not analyze individual parts of the mushrooms, except for several studies that focus on the composition of mycelium. The processing of raw psilocybin mushrooms typically includes agitation, maceration and sonication in methanol [158]. Other solvents or solvent mixtures typically lead to lower extracting efficiencies [159,160,161], which might be due to a hydrolytic (or solvolytic) labile nature of some of the Psilocybin e analogs.

The table below (Table 4) summarizes the species of mushrooms for which the concentrations of the metabolites have been determined. Commonly, psilocybin and psilocin are the quantified metabolites. Throughout the species, their concentration varies from 0.01–3.04% for Psilocybin and 0.0069–0.9% for Psilocin.

The pantropical *P. cubensis* is the most thoroughly characterized species, which can produce, next to the standard alkaloids, also the N,N-diethylamine homologues in relatively high amounts up to 4% combined. These compounds are known to be psychoactive, albeit their affinities towards 5-HT1A are somewhat lower [201]. It is noteworthy that these diethyl analogs are not native to *Psilocybe* mushrooms but were obtained after feeding *P. cubensis* with diethyltriptamine, which could enter the metabolic pathway. Additionally, *P. cubensis* also contains harmane alkaloids in an appreciable amount (up to 0.8%).

Another species that is worth pointing to is *P. semilanceata.* Next to the standard alkaloids, it contains trace amounts of phenylethylamine, which is also psychoactive, but some literature connects it to adverse effects [187]. It is noteworthy that the content of phenylethylamine in this species is in minute amounts; thus, the link between the effects and its presence is questionable.

### 3.6. Criterion 5: Dose Precision and Stability

Tryptamine alkaloid content varies substantially across *Psilocibe* species, and can also vary between individual fruiting bodies. Consequently, dosing based on the dried or fresh mushrooms will be inherently inaccurate. A significantly higher dose precision can be achieved in various extracts.

Crude aqueous extracts (e.g., teas or water infusions) represent the simplest form of extraction. Psilocybin is water-soluble, but psilocin is chemically labile and prone to oxidation and oligomerization, at elevated temperatures and particularly upon exposure to light [197]. As a result, aqueous extracts show low and poorly reproducible extraction efficiency, and their alkaloid content can change over time. Consequently, crude aqueous extracts exhibit low-dose precision and are unsuitable for controlled research or clinical contexts.

Alcoholic extracts, typically prepared using ethanol or methanol, are widely used in analytical chemistry and research settings. Acidic alcoholic solvents improve the solubility and short-term stability of psilocybin and psilocin. Modern liquid chromatography and mass spectrometry methods commonly rely on such extracts for quantification of mushroom alkaloids [202]. When biomass is homogenized and extraction parameters are standardized, alcoholic extracts can achieve moderate dose precision. However, without quantitative analysis, variability in extraction efficiency and matrix effects can still lead to significant uncertainty.

Standardized extracts represent a more controlled approach, in which the concentrations of psilocybin (and often psilocin) are analytically determined, and the extract is normalized to a defined alkaloid content. Studies and regulatory reports emphasize that such standardization, combined with validated analytical methods, substantially reduces variability compared to whole-mushroom biomass. Although minor alkaloids remain present, the primary source of uncertainty shifts from biological variability to analytical measurement error and chemical stability, particularly of psilocin. These extracts therefore offer high-dose precision relative to crude preparations, though they are still inferior to pure drug substances [202,203].

An alternative to liquid means of dosing is standardized products containing solid biomass. It has been demonstrated that under carefully controlled growth conditions, followed by homogenization, good consistency in the content of Psilocybin and psilocin can be achieved [204]. The corresponding product could be stored for 11 months without any notable deterioration. In this case, the adherence to the regulatory criteria was further supported by microbial, pesticide and heavy metal analysis. However, the authors also observed several anomalous batches where the content of psychoactive compounds could be as low as 0.66% and as high as 1.54%. This translates to a 6 g dose of the biomass, showing variation from 19.8 mg to 46.2 mg in the content of the alkaloids. As a result, a 70 kg person could, after using this product, perceive a broad range of experiences from moderate psychedelic effects to full ego dissolution experiences.

Purified psilocybin, either natural or synthetic, provides the highest achievable dose precision. Clinical trials of psilocybin-assisted therapy mainly employ chemically pure psilocybin to ensure milligram-accurate dosing and reproducibility. Once purified, the origin of the molecule (natural vs. synthetic) is completely irrelevant from a dosing standpoint. This approach eliminates variability arising from extraction efficiency, matrix effects, and minor alkaloids.

#### Chemical Stability

Despite being related, psilocybin and psilocin substantially differ in their chemical stability, and this difference underlies most of the degradation observed in mushroom biomass and extracts. Psilocybin is a zwitterionic phosphate ester that is relatively stable in solid form. Psilocin contains a free phenolic hydroxyl group on an electron-rich indole system, rendering it significantly more susceptible to chemical degradation.

The most fundamental transformation affecting psilocybin is dephosphorylation to psilocin. This reaction involves hydrolytic cleavage of the phosphate ester at the 4-position and can occur enzymatically or chemically under acidic or basic conditions. Moisture, elevated temperature, and extreme pH all promote this process, resulting in the conversion of a comparatively stable compound into one that is chemically fragile, thereby accelerating overall potency loss. This is implicitly documented in stability and analytical studies that report changing psilocybin/psilocin ratios depending on sample handling and extraction conditions [202].

Psilocin undergoes oxidative degradation, which constitutes the dominant pathway of irreversible decomposition. The phenolic hydroxyl group at the 4-position is particularly prone to one-electron oxidation, leading to the formation of phenoxyl radical intermediates. These radicals can undergo coupling, rearrangement, or polymerization reactions, producing a mixture of oxidized products that are often darkly colored and show low solubility. This chemistry is consistent with the well-known blue or blue-green discoloration observed when psilocybin mushrooms are bruised or damaged [205].

Following initial phenolic oxidation, psilocin may undergo further degradation involving oxidation of the indole ring itself. These secondary processes can lead to ring opening and fragmentation, generating low-molecular-weight products that lack psychoactive properties and are rarely characterized in detail.

Photodegradation represents an additional accelerant of both psilocybin and psilocin breakdown. The indole chromophore absorbs ultraviolet and visible light, and photoexcitation increases susceptibility to oxidation. Improved stability under light-protected conditions has been demonstrated experimentally for aqueous standard solutions, highlighting the importance of excluding light during storage and analysis [197].

In whole-mushroom material, these chemical pathways are compounded by matrix effects. Psilocybin and psilocin coexist with water, enzymes, metal ions, and a wide range of endogenous metabolites, creating a chemically complex environment. Cell damage, such as that caused by freezing fresh material, can increase contact between enzymes and substrates, enhancing dephosphorylation and oxidation. As a result, degradation in biomass often follows non-linear and poorly predictable kinetics, in contrast to the behavior of isolated compounds under controlled laboratory conditions.

Taken together, the decomposition of psilocybin and psilocin follows the following sequence: psilocybin undergoes dephosphorylation to psilocin, psilocin undergoes phenolic oxidation to radical intermediates, and these intermediates ultimately form non-psychoactive, typically oxidized products. Light, heat, oxygen, moisture, and biological matrix components have an influence on these processes.

### 3.7. Criterion 6: Ecological Sustainability

Cultivating psilocybin-containing mushrooms (e.g., *Psilocybe cubensis*) presents both opportunities and challenges from an ecological and practical perspective. Unlike autotrophic crops that require arable land and direct sunlight, these fungi are primarily saprotrophic decomposers that can be cultivated using diverse organic substrates such as manure, wood debris, soil or even agricultural waste products [13]. In addition, the by-products from mushroom cultivation have downstream potential uses including repeated substrate cycles, animal feed and renewable energy production [206]. This metabolic profile allows for integration into circular agricultural economies, as cultivation can occur in non-arable, controlled indoor environments using substrates that would otherwise be considered waste streams [207].

Despite its practical viability, the cultivation of psilocybin containing mushrooms faces at least two significant ecological and operational challenges. First, mature mushrooms produce enormous quantities of spores. In closed indoor environments, these can cause respiratory health hazards such as asthma or hypersensitivity pneumonitis for cultivators and cause physical pollution within facilities [208]. Spore dispersal from cultivars into the surrounding environment also risks depleting the genetic diversity of wild populations [209].

Second, cultivated mushrooms require specific conditions, such as precise temperature control and humidity, making the process energy-consuming and labor-intensive [210]. Additionally, the nutrient-rich substrates required for *Psilocybe* species are highly susceptible to contamination by competing molds (e.g., *Trichoderma* spp.) and bacteria. Mitigating this requires energy-intensive sterilization or pasteurization processes (autoclaving) and the maintenance of sterile laboratory conditions to prevent crop failure [211]. Consequently, the sustainability of cultivation is heavily dependent on the energy source used for climate control and sterilization, rather than the biological limitations of the fungi themselves.

Foragers of wild psilocybin mushrooms face a different set of challenges, driven largely by environmental instability. In a striking example, Haro-Luna et al. [105] documented shifting practices around *Psilocybe zapotecorum* in Zapotec communities in Oaxaca, Mexico. This community has a long cultural history of use of psilocybin containing mushrooms. These are traditionally consumed in nocturnal rituals and used for guidance, illness insight, and emotional healing. However, Haro-Luna et al.’s ethnographic research highlighted a sharp decline in interest in these practices by younger generations linked to decreased availability of wild foraged mushrooms due to changing climate patterns. Participants reported that these mushrooms are increasingly difficult to find due to reduced precipitation and deforestation. This is consistent with findings from Procházka et al. (2023) [212] who showed that a one unit increase in precipitation corresponded to a 27-tonne increase in culinary foraged mushrooms in Europe.

On balance, prospects for the cultivation of psilocybin containing mushrooms are promising, however there are important environmental impacts that must be carefully managed. At the same time, psilocybin containing mushroom species grow naturally around the world, and wild foraging of these is a viable access pathway in many places (taking into account risks related to identification outlined in Section 3.6). At a global level, changes in climate are likely to result in both decreased and increased availability of wild mushrooms across different bioregions. Nevertheless, these changes are significant as they are likely to increase the loss of Indigenous local knowledge systems associated with ritualistic use of psilocybin containing mushrooms [213].

## 4. Discussion

### 4.1. Critical Assessment of Current Evidence

**Figure 3:**
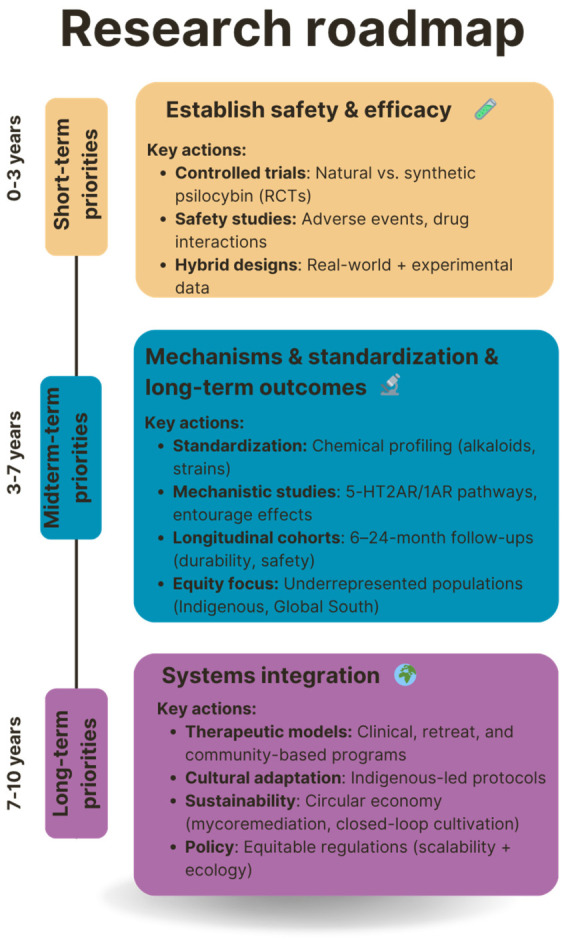
Phased research roadmap for advancing naturally derived psilocybin as a therapeutic and sustainable tool.

The reviewed evidence demonstrates that naturally derived psilocybin is in use with substantial prevalence and consistent well-being-oriented motivations across multiple regions. Population-level analyses from the United States indicate widespread lifetime exposure and increasing use over time [9,33], while large international surveys show psilocybin to be the most commonly used psychedelic globally, primarily for personal growth, psychological well-being, and self-treatment rather than recreation alone [35]. To synthesize the diverse findings in this review, we apply a consistent structure across the six criteria. For each, we summarize: (1) core takeaways and degree of evidentiary support, (2) research gaps (methodological or conceptual limitations), (3) next steps (priorities for future research and application). We then formulate a synthesis across the criteria, which addresses the key research questions, formulate a suggested roadmap for future research (Figure 3), and finish with benefit–risk considerations for policy and therapeutic development. Findings are summarized in Table 5.

#### 4.1.1. Criterion 1: Human Evidence of Therapeutic Benefit

##### Summary Conclusions

The reviewed evidence indicates that naturally derived psilocybin is associated with perceived therapeutic benefits across multiple biopsychosocial domains, including mood, anxiety, trauma-related symptoms, chronic pain, ADHD symptoms, and psychological flexibility [49,50,52,53,56,59]. Evidence is strongest in observational and longitudinal designs and weakest in controlled trials. Benefits appear context-sensitive and moderated by intention, cultural meaning, and social conditions. While causality remains unproven, the convergence of findings across large samples and diverse settings supports therapeutic plausibility and justifies further controlled investigation.

##### Evidence Gaps

Despite encouraging signals, significant gaps remain:

1. Lack of causal evidence: Most studies rely on RWE, e.g., self-report and observational designs [50,53,60], and nearly all clinical trials use synthetic psilocybin. Naturally derived psilocybin remains underexamined in experimental settings [79,80].
2. Underrepresentation of marginalized populations: Differential outcomes by ethnicity and structural context are evident but underexplored [48,52].
3. Heterogeneity of dosing and preparation/limited understanding of dose–response relations: Naturally derived psilocybin varies widely (specifically in microdosing), limiting comparability [57,59,61].
4. Sparse long-term follow-up: Few studies extend beyond six months, leaving questions about the durability and integration of benefits [56].

##### Recommendations for Future Research

- Conduct controlled trials using natural preparations, with transparent reporting following CONSORT-Herbal guidelines (a reporting guideline requiring detailed documentation of botanical identity, preparation, composition, and dosing [30]).
- Integrate real-world evidence with experimental designs, particularly in retreat and self-treatment contexts [50,56].
- Develop culturally sensitive outcome measures that capture well-being, meaning, and social functioning alongside symptom reduction [52].
- Prioritize harm-reduction and safety-informed studies reflecting real-world use patterns [48,58].

#### 4.1.2. Criterion 2: Safety and Tolerability

##### Summary Conclusions

Current evidence indicates that naturally derived psilocybin is generally safe and well tolerated, particularly in intentional and supported contexts [34,39,58,92,93,106]. Adverse effects are typically transient, psychological (e.g., anxiety, confusion, emotional distress), and resolve without intervention, with risk strongly shaped by set, setting, preparation, and supervision [34,39,92,93].

Serious acute harm is rare, with emergency treatment required in fewer than 1% of users; risk increases with younger age, polysubstance use, and unsupervised settings [92]. Cultural context influences interpretation, as experiences classified as adverse in biomedical settings may be viewed as meaningful or therapeutic in ceremonial contexts [89].

The primary medical risks involve drug interactions. SSRIs/SNRIs often attenuate effects, while MAOIs, tricyclics, or stimulants may potentiate physiological responses or rarely cause severe events such as hypertensive crisis [96,97,100].

Natural-source risks include mushroom misidentification, variable potency, and rare syndromes such as wood-lover paralysis, emphasizing the importance of controlled cultivation and screenings [101]. Qualitative evidence highlights trauma-informed preparation and experienced facilitation as key protective factors [39,106].

Overall, psilocybin appears physically safe for most people, and the main risks are reported from controllable factors, such as mixing it with other drugs, unsafe environments, or poor preparation, rather than from the substance itself.

##### Evidence Gaps

1. Lack of controlled safety studies: Most safety data come from observational, self-reported surveys without control groups or standardized dosing, limiting causal conclusions about adverse events, tolerability, and long-term safety [34,50].
2. Limited evaluation of naturally derived psilocybin in rigorous designs: There is a need for controlled and prospective studies specifically examining safety profiles, adverse event rates, and dose–response relationships of naturally derived psilocybin across different use contexts.
3. Real-world harm-reduction for psilocybin use lacks systematic study: Factors like preparation, advice-seeking, and supportive environments are informally linked to safety but remain undefined and unevaluated as formal strategies [39,50,106].
4. Limited understanding of safety determinants in unsupervised use: The mechanisms through which contextual, social, and behavioral factors influence adverse event risk remain insufficiently characterized, particularly in informal or community-based settings.

##### Recommendations for Future Research

- Conduct controlled trials comparing naturally derived psilocybin for physiological and psychological tolerability.
- Develop evidence-based harm-reduction guidance especially for independent users who do not access retreats or clinical settings.

#### 4.1.3. Criterion 3: Psychopharmacological Uniqueness

##### Summary Conclusions

There is minimal data indicating a distinct psychopharmacological profile for naturally derived forms of psilocybin. Only one study has administered both naturally derived and synthetic psilocybin to humans (n = 4), and this study found no difference in clinical or psychological effects [155]. Historical and contemporary reports on the effects of synthetic compared to naturally derived psilocybin similarly report negligible subjective differences. Only two preclinical studies [144,145] have directly compared naturally derived and synthetic psilocybin, and they reported inconsistent findings regarding possible psychological or synaptic effects. There is some preliminary evidence from computational modeling and behavioral animal studies indicating that unpurified mushroom extracts may confer enhanced therapeutic effects via entourage effects. This is based on a small number of unreplicated studies. There is evidence of clear preferences for naturally derived compared to synthetically derived forms of psilocybin, and this preference itself may shape outcomes [151]. Based on current evidence, it is unclear if there is any pharmacological or psychological benefit of naturally derived compared to synthetic forms of psilocybin.

##### Evidence Gaps

1. Direct comparisons: Only one study [155] and limited animal research (e.g., [143]) compare natural vs. synthetic psilocybin with no standardized dosing or blinding.
2. Entourage molecules in humans: No clinical studies have isolated the effects of baeocystin, norbaeocystin, aeruginascin, or β-carbolines, despite preclinical promise.
3. Pharmacokinetic interactions: The metabolic fate of entourage compounds (e.g., MAO inhibition by β-carbolines) remains unquantified in humans.

##### Recommendations for Future Research

- Controlled human trials: Well-powered, double-blind, randomized comparisons of standardized naturally derived and synthetic psilocybin. We can be confident that both arms in such a study would experience broadly similar psychological effects, and so any subtle differences in phenomenology or clinical outcomes at a group level would be revealing.
- Isolation studies of entourage molecules: Investigate baeocystin, norbaeocystin, norpsilocin, aeruginascin in humans (as preclinical data suggests potential antidepressant/MAO-inhibiting properties, e.g., [146,147]), using dose ranges exceeding natural concentrations (to overcome low bioavailability), placebo-controlled design.
- Pharmacokinetic/Pharmacodynamic studies: Quantifying metabolic interactions (β-carboline MAO inhibition), comparing BB penetration and receptor occupancy between naturally derived vs. synthetic forms could explain prolonged effects observed in animal studies [145].

#### 4.1.4. Criterion 4: Identity and Composition Control

##### Summary Conclusions

The chemical characterization of psilocybin-producing mushrooms reveals a complex and taxonomically diverse landscape. Across the genus *Psilocybe*, spanning at least 144 hallucinogenic species distributed globally, psilocybin and psilocin are the dominant psychoactive compounds. Additional constituents include baeocystin, norbaeocystin, aeruginascin, norpsilocin, harmane alkaloids, and phenylethylamine.

The compositional variability across and within species is substantial. Psilocybin concentrations range from 0.01% to 3.04% dry weight, and psilocin from 0.007% to 0.9%, with wide intraspecies variation observed even within well-characterized species such as P. cubensis and P. semilanceata. This variability likely reflects differences in genetic strain, growth substrate, cultivation conditions, geographic origin, and post-harvest handling, none of which are systematically controlled or reported across the literature. Methanol extraction is the established standard, with alternative solvents consistently yielding lower efficiency, possibly due to the hydrolytic lability of some psilocybin analogs. The chemical composition of natural psilocybin preparations can be reliably characterized and quantified using liquid chromatography methods.

##### Evidence Gaps

1. Contaminant profiling (heavy metals, pesticides, microbial load) is absent from the reviewed literature, despite being required under EMA and WHO GACP guidelines for any herbal medicinal product.

##### Recommendations for Future Research

- Characterize minor psychoactive constituents pharmacologically, particularly harmane alkaloids and phenylethylamine, to determine whether their concentrations in natural products are clinically meaningful or negligible.
- Analyze mushroom fractions separately (mycelium, stipe, cap, spores) to identify compositional hotspots relevant to extraction design and quality control.

#### 4.1.5. Criterion 5: Dose Precision and Stability

##### Summary Conclusions

Dosing precision in naturally derived psilocybin products faces two core challenges: biological variability and chemical instability.

Biological variability arises from inconsistent alkaloid content across *Psilocybe* species and even within individual fruiting bodies. Psilocybin concentrations can range from 0.2–5.3 mg/g dry weight, a 25-fold variation, making weight-based dosing unreliable for clinical research. Precision improves with processing, from crude extracts (e.g., teas) to standardized extracts and purified psilocybin.

Chemical instability further complicates dosing. Psilocin, the active metabolite, is highly unstable due to its phenolic hydroxyl group, which undergoes oxidative degradation. Psilocybin dephosphorylates into psilocin under hydrolytic conditions, and psilocin oxidizes into non-psychoactive polymers, causing the characteristic blue bruising. Light, heat, enzymes, and physical disruption (e.g., freezing or maceration) accelerate degradation, leading to unpredictable kinetics.

Aqueous extracts are particularly unstable, while alcoholic extracts offer short-term stability. Standardized extracts reduce variability but remain vulnerable to psilocin degradation. Purified psilocybin, whether natural or synthetic, is the only form capable of milligram-accurate dosing, eliminating biological and chemical uncertainties.

Dose precision follows a clear hierarchy: purified psilocybin > standardized extracts > alcoholic extracts > aqueous extracts > whole biomass.

Psilocin is chemically labile and the dominant source of instability across all preparation types. Psilocybin is comparatively stable in solid form but serves as a precursor to psilocin via dephosphorylation.

##### Evidence Gaps

1. Systematic stability data for standardized extracts under defined storage conditions (temperature, humidity, light) are lacking.
2. Degradation kinetics in biological matrices are poorly characterized quantitatively; most reports describe the direction of change rather than the rate or predictability of degradation under realistic storage conditions.
3. Minor alkaloid stability—including baeocystin, norbaeocystin, aeruginascin, and harmane alkaloids—has not been systematically studied, yet these compounds may contribute to pharmacological variability as major alkaloids degrade.
4. Aqueous extract behavior at varying temperatures and preparation methods (e.g., short infusion vs. prolonged boiling) has not been systematically characterized in terms of alkaloid yield and degradation products.
5. Freeze–thaw effects on fresh mushroom material are mentioned as accelerating enzymatic degradation, but controlled studies quantifying the magnitude of this effect on final alkaloid content are absent.
6. Oxidation product characterization is not complete yet—the ring-opening and fragmentation products of psilocin are rarely identified or quantified, leaving the full profile of degradation byproducts unknown.
7. There is currently no published formal stability study comparing natural and synthetic purified psilocybin under equivalent conditions to determine whether origin affects shelf life or degradation profile.

##### Recommendations for Future Research

- Characterize oxidative degradation products of psilocin systematically to assess whether any byproducts carry residual pharmacological or toxicological activity.
- Investigate minor alkaloid degradation in parallel with major alkaloid studies to determine whether differential stability alters the pharmacological fingerprint of extracts over time.

#### 4.1.6. Criterion 6: Ecological Sustainability

##### Summary Conclusions

The cultivation of psilocybin-containing mushrooms presents an ecologically innovative model, leveraging waste substrates and controlled environments to integrate into circular agricultural systems. However, the process is not without trade-offs, as energy-intensive sterilization and climate control requirements challenge its sustainability. Simultaneously, wild foraging, while culturally significant, faces disruption due to environmental shifts, threatening both mushroom availability and Indigenous knowledge systems. Together, these findings suggest that while controlled cultivation may offer a scalable solution, its ecological footprint and the preservation of wild populations must be carefully balanced.

##### Evidence Gaps

1. Key gaps remain in understanding the long-term ecological impacts of large-scale psilocybin mushroom cultivation, particularly regarding spore dispersal and genetic diversity.
2. Research on less energy-intensive sterilization methods could improve sustainability.
3. The effects of climate change on wild psilocybin species distribution are also understudied, particularly in regions where traditional foraging is culturally significant.

##### Recommendations for Future Research

- Future studies should prioritize low-energy cultivation methods, such as passive climate control or alternative sterilization processes, to improve sustainability.
- Investigating spore mitigation strategies, including filtration systems or genetic modification to reduce sporulation, could address health and ecological concerns.
- Ethnographic and ecological research should also focus on documenting Indigenous knowledge systems and identifying climate-resilient foraging practices.
- Interdisciplinary collaboration between mycologists, agricultural scientists, and Indigenous stakeholders could yield more holistic approaches to psilocybin mushroom production.

### 4.2. Synthesis: Addressing the Key Research Questions

1. Do naturally derived psilocybin preparations demonstrate therapeutic potential in human populations (in real-world use)? (T)

Multiple observational studies, including REW, report consistent associations between natural psilocybin use and improved mental health outcomes, including reductions in depression/anxiety and enhancements in well-being. While these findings suggest therapeutic potential, they are limited by non-causal designs (e.g., lack of randomization, placebo controls) and cannot isolate psilocybin’s effects from contextual factors (e.g., set/setting, expectancy). Thus, preliminary evidence is promising, but controlled trials are needed to confirm efficacy and safety in clinical populations.

2. Do naturally derived psilocybin preparations produce pharmacological or experiential effects that are meaningfully distinct from synthetic psilocybin? (S)

Current evidence is largely inconclusive: Preclinical data suggest unpurified mushroom extracts may enhance neuroplasticity and synaptic plasticity via entourage compounds, but human validation is absent. Only one small trial directly compared naturally derived vs. synthetic psilocybin, finding subtle subjective differences, but without clear pharmacological distinctions. No studies have isolated the effects of entourage molecules in humans. Surveys show a strong preference for natural psilocybin, but this may reflect cultural biases (e.g., “natural = better”) rather than pharmacological differences. Thus, no empirical evidence currently supports unique pharmacological effects of natural psilocybin, though preclinical mechanisms (e.g., entourage effects) warrant further investigation.

3. Do naturally derived psilocybin preparations meet criteria for consideration as therapeutic agents or regulated medicinal products? (MP)

No. Natural psilocybin preparations fail to meet EMA/FDA standards for medicinal products due to: High biological variability: >25-fold differences in psilocybin content across *Psilocybe* strains/parts (cap vs. stem vs. mycelium); Uncharacterized composition: Contains multiple bioactive compounds (e.g., harmane alkaloids, phenylethylamine) with unknown pharmacological contributions; Lack of standardization: No composition profiling (amino acids, sugars, alkaloids) or stability data, both mandatory for regulatory approval; Dosing imprecision: Variability complicates reproducible clinical use. Thus, only chemically pure psilocybin (synthetic or isolated pure mushroom extracts) currently meets regulatory criteria. Standardization efforts (e.g., strain selection, extraction protocols, quality control) are required before natural preparations can be considered for medicinal approval.

### 4.3. Roadmap for Future Research

To advance naturally derived psilocybin as a therapeutic and culturally integrated tool, research should follow a phased, interdisciplinary roadmap addressing clinical efficacy, mechanistic insights, and systems-level integration.

#### Short-Term Priorities (0–3 Years): Establish Safety and Efficacy

1. **Controlled clinical trials**
• Randomized, double-blind trials of standardized, naturally derived preparations vs. synthetic psilocybin to determine equivalence (clinical outcomes, tolerability, and subjective effects) and dose-dependent relationships.
2. **Safety evaluation**
• Prospective studies to quantify: adverse events, drug interactions, and contextual risk factors.
3. **Hybrid real-world evidence with experimental designs**
• Hybrid designs combining observational studies with experimental elements could capture ecologically valid contexts while improving causal inference.

Mid-term priorities (3–7 years): Mechanisms and Standardization

1. **Standardization and characterization**
• Chemical profiling of alkaloids (e.g., psilocybin, baeocystin, β-carbolines) across species, strains, cultivation methods, and processing conditions, adopting CONSORT-Herbal guidelines for transparency.
2. **Mechanistic studies**
• Dose–response relationships Research should clarify relationships between dose, subjective experience (e.g., mystical-type experiences), neurobiological mechanisms, and clinical outcomes.
3. **Cultural and equity research**
• Inclusive cohorts to examine structural moderators (e.g., chronic stress, discrimination), Indigenous and marginalized perspectives.
4. **Longitudinal outcome studies**
• Prospective cohorts with follow-ups to evaluate the durability of therapeutic effects, long-term safety (psychiatric stability, neurocognitive effects, behavioral changes), and contextual moderators (e.g., clinical vs. retreat vs. home use, social support).
• Naturalistic longitudinal designs: GPS follow-ups or registry studies to capture real-world trajectories.

#### Long-Term Priorities (7–15 Years): System Integration

1. **Therapeutic and cultural implementation**
• Delivery models: Studies should evaluate safe delivery models across clinical settings, structured retreat environments, and community-based harm-reduction or integration programs.
• Regulatory pathways with adaptive licensing (e.g., “botanical drug”).
• Community-led protocols for traditional and modern use.
2. **Ecological sustainability**
• Circular economy models: Integrate mycoremediation and optimize closed-loop cultivation.
• Biodiversity conversion: Establish protected foraging zones and seed/spore bands for wild strains.
• Policy and stakeholder collaboration: Advocate for regulations balancing scalability with ecological/social equity, including interdisciplinary working groups.

## 5. Conclusions

Taken together, the available evidence suggests that naturally derived psilocybin presents a favorable but still preliminary benefit–risk profile. Observational and naturalistic studies across large and diverse populations consistently report perceived improvements in psychological well-being, mood, trauma-related symptoms, and personal growth, particularly when use occurs in intentional, supportive contexts. Adverse effects appear generally transient and primarily psychological, although these may be under-reported. Identified risks are largely context-dependent and modifiable, including polysubstance use, unsupervised settings, inappropriate dosing, and drug interactions.

However, the current evidence base is dominated by real-world observational data, with relatively few controlled trials using naturally derived preparations. Consequently, while the convergence of findings across epidemiological surveys, prospective naturalistic studies, and qualitative research supports therapeutic plausibility, causal effects and optimal safety parameters remain insufficiently established.

From a regulatory perspective, these findings suggest that naturally derived psilocybin warrants carefully regulated research and development pathways rather than categorical prohibition, particularly given the increasing prevalence of real-world use. Policy frameworks that facilitate controlled clinical studies, standardized cultivation and preparation practices, and evidence-based harm-reduction approaches could help clarify therapeutic potential while minimizing risks. Importantly, current evidence does not support meaningful pharmacological differences between naturally derived and synthetic psilocybin, suggesting that regulatory considerations should focus primarily on standardization, safety, and context of use.

Overall, the existing literature indicates that naturally derived psilocybin represents a promising but still investigational therapeutic candidate, with a risk profile that appears manageable under appropriate safeguards. Advancing the evidence base through rigorous trials, standardized reporting, and integration of real-world evidence will be essential to inform balanced policy and drug development decisions.

## Figures and Tables

**Table 1:** Six-criterion framework to evaluate naturally derived psilocybin.

Criterion	Leading Question	Description	T	S	MP
1. Human evidence of therapeutic benefit	Does naturally derived psilocybin demonstrate measurable therapeutic outcomes in real-world and clinical populations?	**Formal evidence**: Controlled trials (RCTs, double-blind) and experimental human studies, measuring efficacy via validated biomedical (e.g., neuroimaging, biomarkers) and psychological (e.g., psychometric scales for mood, anxiety, post-traumatic stress disorder) outcomes. **RWE**: Observational studies, naturalistic studies, field/lab studies, longitudinal cohorts, surveys, Indigenous/community-based practices, and practitioner case reports.	✔		✔
2. Safety and tolerability	What is known about adverse events, contraindications, and the contextual factors influencing risk and tolerability?	**Formal evidence**: Preclinical: toxicology, animal models, pharmacokinetics. Early phase human studies: Controlled safety/tolerability trials (healthy participants), clinical safety data, adverse event reporting, pharmacovigilance systems. **RWE**: Survey data on adverse effects, naturalistic studies, field/lab studies, Indigenous harm-reduction practices, community monitoring, and practitioner case reports.	✔		✔
3. Unique pharmacological profile (vs. synthetic)	Do naturally derived psilocybin preparations contain unique compounds or synergistic profiles meaningfully distinct from synthetic psilocybin?	**Formal evidence**: Comparative pharmacokinetic/pharmacodynamic studies, chemical profiling, bioassay tests for synergistic effects. **RWE**: User reports, naturalistic studies, field/lab studies, and Indigenous/community-based observation of experiential differences.		✔	✔
4. Identity and composition control	Can naturally derived psilocybin be reliably identified, authenticated, and shown to be free from harmful contaminations?	**Formal evidence**: Laboratory authentication, chemical fingerprinting, contaminant testing (EMA/HMPC standards) **RWE**: Community/practitioner-reported sourcing, ethnobotanical classification systems. Supply chain tracking	✔	✔	✔
5. Dose precision and stability	Are naturally derived psilocybin preparations consistent in potency and stable over time, allowing accurate and reproducible dosing?	**Formal evidence**: Quantitative assays for stable psilocybin/psilocin content under defined storage (temperature, humidity, light). **RWE**: User-reported dosing practices, ceremonial norms, observational stability, incl. measurement methods and environmental factors	✔	✔	✔
6. Ecological sustainability	Can naturally derived psilocybin be sourced or cultivated at scale without ecological harm or cultural disruption?	**Formal evidence**: Ecological impact studies, biodiversity audits, and sustainable yield modeling. **RWE**: Indigenous ecological knowledge, community observations of habitat changes, and seasonal availability.	✔		✔

Note. For each criterion, a leading question to frame its purpose is included, as well as a description of the types of evidence. The column and checkmarks T, S, and MP indicate which of the three main research questions each criterion addresses. T: Therapeutic potential, S: Specificity, MP: Medicinal product. The six criteria (Table 1) were developed based on established regulatory and consensus frameworks, including CONSORT-Herbals, FDA Botanical Guidance, EMA/HMPC, and WHO GACP (see References [28,30,31,32]). RWE: Real-world evidence.

**Table 2:** Overview of psilocybin-related tryptamine alkaloids.

Name	Chemical Structure	Role
Psilocybin	4-phosphoryloxy-N,N-dimethyltryptamine	Dephosphorylated into psilocin
Psilocin	4-hydroxy-N,N-dimethyltryptamine	Activates 5-HT_2A_ receptors; alterations in consciousness; physiological changes
Baeocystin	4-phosphoryloxy-N-methyltryptamine	Dephosphorylated into norpsilocin
Norpsilocin	4-hydroxy-N-methyltryptamine	Activates 5-HT_2A_ receptors (in vitro) *
Norbaeocystin	4-phosphoryloxytryptamine	Dephosphorylated form activates 5-HT_2A_ receptors
Aeruginascin	4-hydroxy-N,N,N-trimethyltryptamine	Bind 5-HT_1A_, _2A,_ and _2B_ receptors

* Degraded by MAO before psychedelic effects can occur.

**Table 3:** Binding affinities and activation potency at some receptors and transporters (Rickli et al. [118]).

**Receptor**	**5-HT_1A_**		**5-HT_2A_**		**5-HT_2B_**		**5-HT_2C_**	
Receptor binding K_i_ ± SD [µM]	0.123 ± 0.02		0.049 ± 0.01		-		0.094 ± 0.009	
Activation potency EC_50_ [µM]	-		0.721 ± 0.55		>20		-	
**Receptor**	**α_1A_**	**α_2A_**	**D_1_**	**D_2_**		**D_3_**		**H_1_**
Receptor binding K_i_ ± SD [µM]	6.7 ± 1.1	2.1 ± 0.01	>14	3.7 ± 0.6		8.9 ± 0.8		1.6 ± 0.2
**Transporter**	**DAT**		**NET**			**SERT**		
Receptor binding K_i_ ± SD [µM]	>30		13 ± 1.7			6.0 ± 0.3		
IC_50_ [µM] (95% CI)	>100		14 (10–19)			3.9 (3.1–4.8)		

**Table 4:** Most common *Psilocybe* species and their respective composition in relevant alkaloids.

Mushroom Species	Metabolites and Concentrations	References
*P. arcana*	Psilocybin—0.01–1.15% and Psilocin—0.03–0.85%	[162]
*P. argentipes*	Psilocybin—0.12–0.38% and Psilocin—0.0069%	[163,164]
*P. baeocystis*	Psilocybin—0.15–0.85% Psilocin—0.048–0.3%, Baeocystin—0.01–0.1%	[165,166,167,168,169]
*P. bohemica*	Psilocybin—0.31–1.12%, Baeocystin—0.02–0.03%	[170]
*P. cubensis*	Psilocybin—0.01–1.35%, Baeocystin—0.01–0.78%, Norpsilocin—0.01–0.02%, Psilocin—0.01–0.78%, Norbaeocysin 0.01–0.05%, Harmanes < 0.8%	[142,165,169,171,172,173,174,175,176,177,178,179,180,181,182]
*P. cyanascens*	Psilocybin 0.1–1.84%, Baeocystin 0.004–0.04%, Psilocin 0.06–0.76%	[161,162,165,169,171,183,184,185,186]
*P. semilanceata*	Psilocybin—0.02–1.70%, Baeocystin—0.02–1.10%, Psilocin—0.01–0.90%, Norbaeocystin—0.077%, Aeruganascin—0.022%, phenylethylamine—0.00001–0.000145%	[144,161,162,165,169,173,174,175,176,186,187,188,189,190,191,192,193,194]
*P. silvatica*	Psilocybin—0.004–0.02%	[169,195]
*P. subaeruginosa*	Psilocybin—0.45–1.41%, Psilocin—0.011–0.038%	[196,197,198]
*P. subcubensis*	Psilocybin—0.32%, psilocin—0.06	[163]
	Psilocybin—0.80–0.86%, psilocin 0.02–0.03%	[199]
*P. tampanensis*	Psilocybin—0.01–0.19 and psilocin = 0.01–0.03%	[176]
*P. zapotecorum*	Psilocybin—1.06–3.04%, Psilocin 0.03–0.65%, Baocystin 0.0024–0.321%, Norpsilocin 0.024–0.142, Aeruganascin-0.01%, 4-hydroxytryptamine—0.036–0.271%	[200]

**Table 5:** Evaluation summary—naturally derived psilocybin.

Criterion	Key Findings	Key Gaps	Recommended Next Steps	Evaluation
1. Human evidence of therapeutic benefit	Consistent perceived improvements across multiple biopsychosocial domains (e.g., mood, anxiety, trauma-related symptoms) [observational and longitudinal]	Causality unconfirmed; limited controlled trials	Conduct controlled double-blind studies comparing naturally derived vs. synthetic psilocybin.	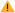 Promising but unconfirmed
2. Safety and tolerability	Generally safe; transient psychological effects, rare serious harm (<1%). Risks: drug interaction, misidentification, context	Safety evidence is mostly observational/self-reported; limited controlled studies on natural preparations.	Prospective safety studies; develop evidence-based harm-reduction guidelines for real-world use.	✅ Mostly favorable
3. Unique pharmacological profile	No clear evidence of unique effects (naturally derived vs. synthetic). Survey preference for naturally derived forms.	No human trials on entourage effects; lack of head-to-head comparisons with synthetic psilocybin.	RCTs investigating direct/synergistic effects of entourage molecules.	❌ Not met
4. Identity and composition control	Composition reliably characterized via liquid chromatography.	Contaminant profiling (heavy metals, pesticides, microbial load) absent; minor constituents uncharacterized.	Pharmacologically characterize minor psychoactive constituents for clinical meaningfulness; analyze mushroom fractions separately	✅ Partially met
5. Dose precision and stability	Dose precision hierarchy: purified > standardized extracts > alcoholic > aqueous > whole biomass. Psilocin is labile; psilocybin is stable in solid form.	Lack of stability data for extracts; degradation kinetics poorly characterized; minor alkaloid stability unstudied.	Systematically study stability under defined conditions; characterize oxidative degradation products.	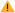 Not met yet
6. Ecological sustainability	Promise as a circular agriculture model.	Energy/climate control challenges; climate change threats to Indigenous practices; long-term ecological impacts unknown.	Investigate low-energy cultivation; develop spore mitigation; support Indigenous knowledge systems.	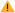 Partially met

## Data Availability

No new data were created or analyzed in this study, Data sharing is not applicable.
